# Antioxidant Carbon Dot of Selenomethionine Alleviates Oxidative Stress in Intervertebral Disc Degeneration

**DOI:** 10.1002/advs.202514217

**Published:** 2025-09-17

**Authors:** Qingzheng Zhang, Zongtai Liu, Yirong Sun, Changfeng Fu, Jianxun Ding

**Affiliations:** ^1^ Department of Spine Surgery, Center of Orthopedics The First Hospital of Jilin University 1 Xinmin Street Changchun 130061 P. R. China; ^2^ State Key Laboratory of Polymer Science and Technology Changchun Institute of Applied Chemistry Chinese Academy of Sciences 5625 Renmin Street Changchun 130022 P. R. China; ^3^ School of Applied Chemistry and Engineering University of Science and Technology of China 96 Jinzhai Road Hefei 230026 P. R. China

**Keywords:** alleviation of intervertebral disc degeneration, attenuation of oxidative stress, carbon dot, nanozyme, scavenging of reactive oxygen species

## Abstract

Intervertebral disc degeneration (IVDD) is a progressive degenerative disorder of the spine characterized by oxidative stress and cellular dysfunction. Current clinical treatments for IVDD include surgical procedures, such as discectomy and spinal fusion, as well as pharmacological therapies using analgesics and anti‐inflammatory agents. However, these strategies primarily offer symptomatic relief and do not address the underlying causes of IVDD. Targeting oxidative stress has emerged as a promising therapeutic approach, yet its effectiveness remains limited. To overcome this limitation, an antioxidant carbon dot of selenomethionine (Se‐Met‐CD) is developed for efficient IVDD therapy. Se‐Met‐CD effectively scavenges excess reactive oxygen species (ROS), alleviates oxidative stress, and restores intracellular redox homeostasis in nucleus pulposus cells. Se‐Met‐CD exhibits an antioxidant capacity 3.1 times that of its precursor, Se‐Met, when compared at the same concentration. In hydrogen peroxide (H_2_O_2_)‐treated nucleus pulposus cells, Se‐Met‐CD significantly reduces intracellular ROS levels to 17.7% of those in untreated control cells. In a puncture‐induced IVDD rat model, Se‐Met‐CD demonstrates remarkable therapeutic efficacy by significantly attenuating disc degeneration, reflected in lower Pfirrmann and histological scores. These results underscore the potential of Se‐Met‐CD to treat IVDD by scavenging ROS, restoring antioxidant balance, and modulating the local microenvironments.

## Graphical abstract

A selenomethionine‐derived carbon dot (Se‐Met‐CD) serves as a potent nano‐antioxidant, efficiently scavenging reactive oxygen species, protecting nucleus pulposus cells from oxidative damage, restoring extracellular matrix homeostasis, and significantly mitigating intervertebral disc degeneration in vivo, which demonstrates great promise as a nanozyme‐based therapeutic strategy for treating degenerative spinal disorders through redox modulation.

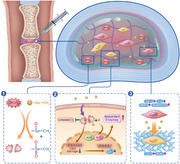

## 1 Introduction

Intervertebral disc degeneration (IVDD) is a prevalent clinical condition marked by degenerative changes in the spine, serving as the pathological basis of disorders, such as disc herniation, degenerative lumbar spondylolisthesis, and spinal canal stenosis.^[^ ^1^, ^2^ ^]^ IVDD is a leading cause of low back pain and results in progressive motor impairment and substantial disability in advanced cases.^[^ ^3^ ^]^ The pathogenesis of IVDD involves a multifaceted cascade initiated by the loss of viability and function in nucleus pulposus cells (NPCs).^[^ ^4^ ^]^ This cellular dysfunction leads to a hostile microenvironment characterized by elevated oxidative stress, chronic inflammation, and disrupted extracellular matrix (ECM) metabolism, ultimately causing progressive structural damage to the intervertebral disc.^[^ ^5^, ^6^ ^]^ Globally, low back pain affects ≈623 million people, with IVDD being a primary cause in ≈40% of cases.^[^ ^7^, ^8^ ^]^ Current clinical management includes surgical procedures, such as total disc replacement, intervertebral fusion, and partial discectomy, along with pharmacological therapies involving non‐steroidal anti‐inflammatory drugs, analgesics, and muscle relaxants.^[^ ^9^, ^10^ ^]^ However, these interventions mainly relieve symptoms and do not halt disease progression.^[^ ^11^ ^]^ Therefore, there is an urgent unmet need for therapeutic strategies to disrupt the degenerative cycle of IVDD and restore homeostasis within the disc.

Numerous therapeutic strategies have been explored in basic research settings to slow the progression of IVDD, including the mitigation of oxidative stress, inhibition of catabolic activity, suppression of inflammation, stimulation of anabolic processes, prevention of cellular senescence, inhibition of apoptosis, and the development of refined biotherapies.^[^ ^12^, ^13^, ^14^ ^]^ Among these, antioxidant‐based therapies have shown considerable promise due to their ability to alleviate oxidative stress.^[^ ^15^ ^]^ For example, Huang et al. demonstrated that isoliquiritigenin effectively reduces reactive oxygen species (ROS) levels and prevents mitochondrial dysfunction in NPCs, thereby attenuating IVDD.^[^ ^16^ ^]^ However, conventional antioxidants often exhibit limited efficacy due to their insufficient capacity to scavenge ROS. In this context, advanced nanomaterials with intrinsic antioxidant properties offer a compelling alternative.

**Scheme 1:**
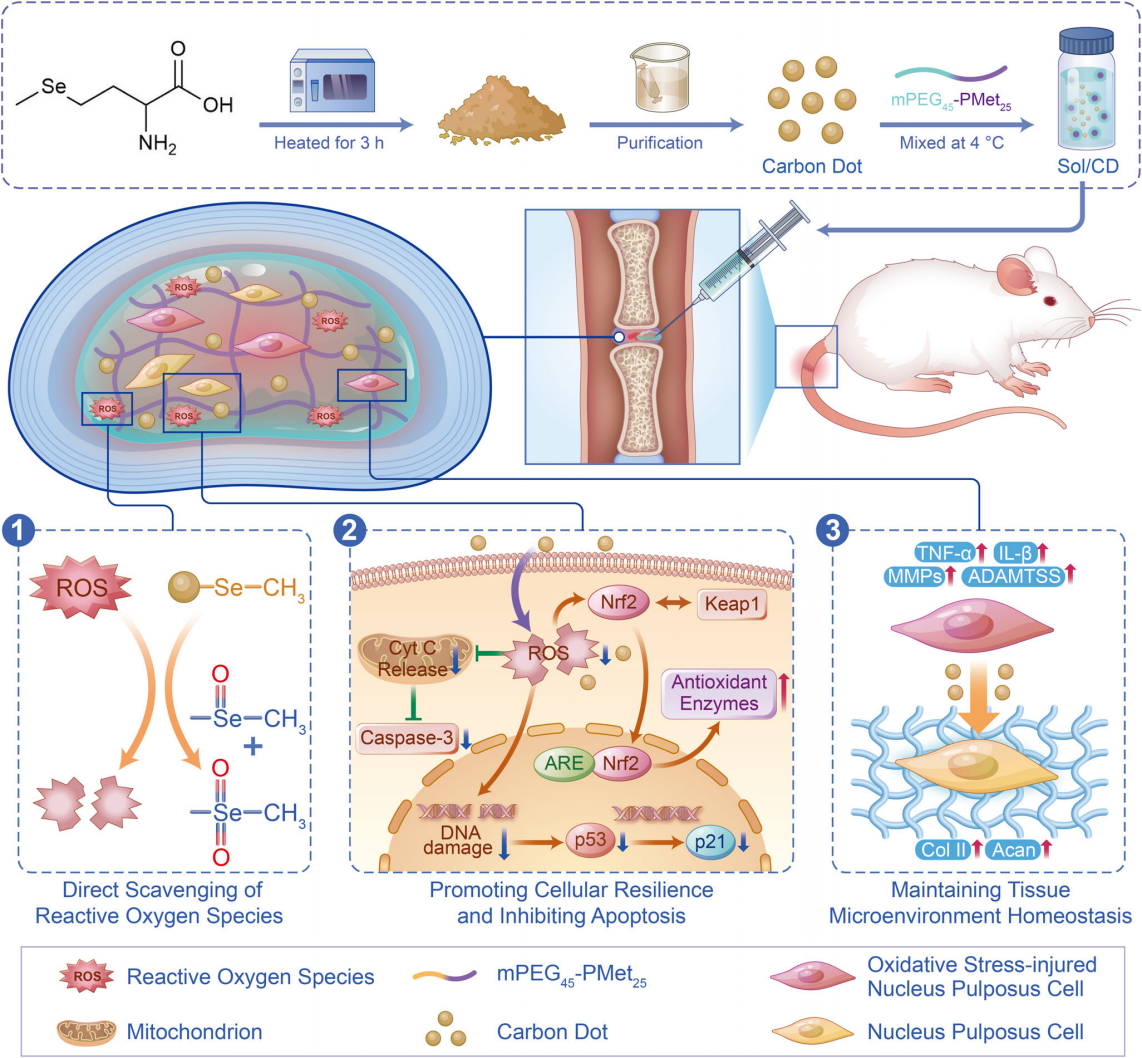
Selenium‐doped CD, delivered by a thermo‐sensitive hydrogel, which directly scavenges ROS, combats oxidative stress, promotes cellular resilience, inhibits apoptosis, and restores matrix homeostasis in intervertebral disc degeneration, effectively attenuating disease progression in a rat model.

Here, carbon dot (CD)‐based antioxidants were designed and utilized to investigate the critical influence of selenium (Se) as compared to sulfur (S). Using selenomethionine (Se‐Met) and methionine (Met) as single precursors, this investigation revealed that Se‐doping substantially enhances the antioxidant performances of CDs. The resulting Se‐Met‐CD protects NPCs from oxidative damage, preserves ECM homeostasis in vitro, and markedly alleviates IVDD progression in a rat model (**Scheme** **1**). These findings highlight the potential of Se‐Met‐CD as a therapeutic nanomaterial capable of simultaneously neutralizing oxidative stress and restoring disc homeostasis in IVDD.

## 2 Results and Discussion

### 2.1 Synthesis and Characterizations of Se‐Met‐CD

CDs are emerging zero‐dimensional carbon nanomaterials with strong potential in biomedicine due to their excellent biocompatibility, facile synthesis, and tunable optical properties.^[^ ^17^, ^18^, ^19^ ^]^ Importantly, their intrinsic biological activities can be precisely tailored via precursor selection and heteroatom doping.^[^ ^20^ ^]^

A key objective of this study was to systematically investigate and isolate the specific contribution of Se doping to the therapeutic performance of CDs. To achieve this, three distinct types of CDs were synthesized and compared using valine (Val), Met, and Se‐Met as precursors. The selection of Val and Met as controls was a deliberate choice rooted in their significant structural analogy to Se‐Met.^[^ ^21^ ^]^ Specifically, all three amino acid precursors possess a comparable number of carbon atoms, ensuring a consistent carbon backbone for the resulting CDs. Furthermore, they are all neutral amino acids characterized by nonpolar, hydrophobic side chains, which lack additional charged functional groups.^[^ ^22^ ^]^ This carefully designed comparative framework is crucial as it minimizes potential variations in the fundamental physicochemical properties of CDs that could arise from structurally different precursors. Consequently, the superior antioxidant properties and enhanced therapeutic efficacy against IVDD observed in Se‐Met‐CDs can be confidently attributed primarily to the specific role of doped selenium, rather than to other confounding structural factors.

In the present work, Se‐Met‐CD, Met‐CD, and Val‐CD were synthesized from their respective precursor amino acids using a simple one‐step thermal method. The optimal synthesis temperatures were determined through iterative experiments to be 160 °C for Se‐Met, 180 °C for Met, and 220 °C for Val, corresponding to the highest production yields of each type of CD. Under these conditions, the fluorescence quantum yields of Se‐Met‐CD, Met‐CD, and Val‐CD were 5.4%, 7.1%, and 12.1%, respectively. Because bond dissociation is typically the rate‐limiting step in pyrolysis and carbonization during the formation of CDs, variations in optimal temperatures likely reflect differences in bond dissociation energy and side‐chain reactivity. Val contains stable C─C and C─H bonds with higher dissociation energies than the C─S bond in Met, which in turn is more stable than the C─Se bond in Se‐Met. The greater reactivity of Se and S allowed the degradation and polymerization to occur at lower temperatures in Met and Se‐Met compared to Val. These findings align with reported thermal stability trends for Se‐ and S‐containing compounds under hydrothermal conditions.^[^ ^23^, ^24^ ^]^

**Figure 1:**
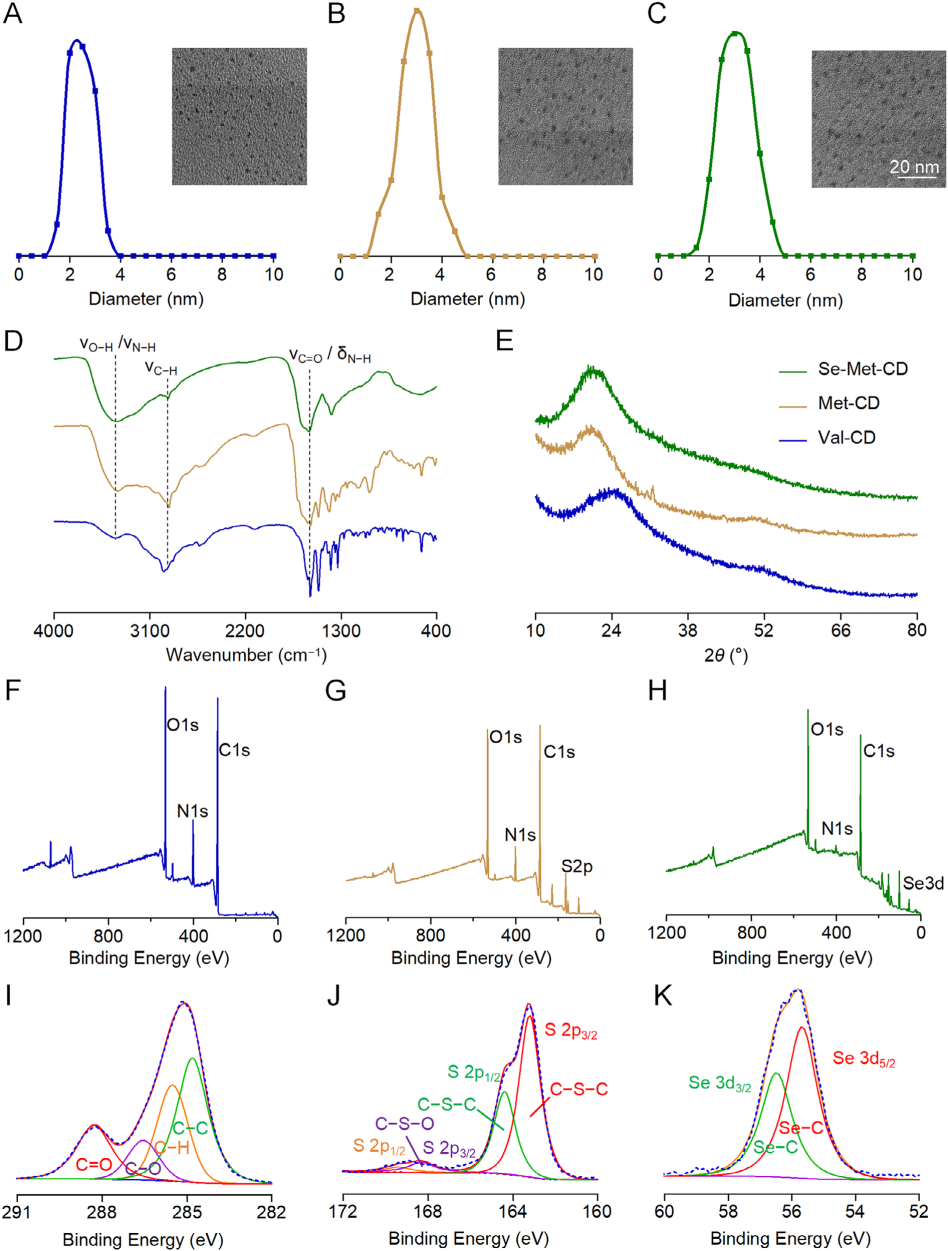
Physical and chemical structures of Val‐CD, Met‐CD, and Se‐Met‐CD. A─C) Particle size distributions and corresponding transmission electron microscopy images for Val‐CD A), Met‐CD B), and Se‐Met‐CD C) (scale bar: 20 nm). D,E) FT‐IR spectra D) and X‐ray diffraction patterns E) for CDs. F─H) Full‐scan XPS survey spectra for Val‐CD F), Met‐CD G), and Se‐Met‐CD H). I) High‐resolution C 1s XPS spectrum for Val‐CD. J) High‐resolution S 2p XPS spectrum for Met‐CD. K) High‐resolution Se 3d XPS spectrum for Se‐Met‐CD. Val‐CD is represented in blue, Met‐CD in brown, and Se‐Met‐CD in green.

The morphologies and sizes of as‐synthesized CDs were examined using transmission electron microscopy (TEM). The average particle sizes were 2.44 nm for Val‐CD (**Figure** **1**A), 2.94 nm for Met‐CD (Figure 1B), and 3.08 nm for Se‐Met‐CD (Figure 1C). The similar sizes likely resulted from the consistent synthesis method and the structural resemblance among the precursor amino acids.

To investigate the structural transformations during synthesis, the chemical structures of CDs (Figure 1D) and their precursors (Figure S1, Supporting Information) were analyzed using Fourier transform‐infrared (FT‐IR) spectroscopy. The FT‐IR spectra exhibited absorption peaks at 3425 cm^─1^, which were attributed to the stretching vibrations of N─H and O─H bonds. Peaks at 2968 and 2880 cm^−1^ corresponded to the stretching vibration of C─H bond, and the peak at 1587 cm^−1^ was assigned to the stretching vibration of carboxyl C═O bond. The peaks at 1510 and 1395 cm^−1^ were attributed to bending vibrations of N─H and C─H bonds, respectively. CDs and their corresponding precursors showed infrared absorption peaks in essentially the same positions, but the pyrolyzed products exhibited weaker intensity, indicating that some bonds of the precursor amino acids had been broken, and the number of functional groups had been reduced. These changes confirmed the conversion of amino acids to the carbon‐based nanostructures (Figure 1D).

To confirm the formation of a graphitic carbon framework, a characteristic feature of CDs, X‐ray diffraction (XRD) analysis was conducted. All three types of CDs exhibited a broad, weak diffraction peak between 20° and 30° (2θ), corresponding to the (002) plane of graphitic carbon (Figure 1E). This indicated successful synthesis of CDs. The peak for Val‐CD appeared at 24.7°, whereas Met‐CD and Se‐Met‐CD exhibited peaks at 19.3° and 19.6°, respectively. The proximity of peaks for Met‐CD and Se‐Met‐CD may be ascribed to the high structural similarity between their precursor amino acids, which differ by only one atom. For comparison, the precursor amino acids were also analyzed by XRD (Figure S2, Supporting Information).

The surface elemental composition and chemical bonding states of CDs were further investigated using X‐ray photoelectron spectroscopy (XPS). Survey scans confirmed that the chemical composition of each CD type closely reflected the structure of its respective precursor amino acid (Figure 1F–H), and high‐resolution spectra identified the specific chemical states of key elements (Figure 1I–K).

For the Val‐CD (Figure S3, Supporting Information), no S or Se signals were detected, confirming the absence of heteroatom contamination. The high‐resolution C 1s spectrum was deconvoluted into four peaks, corresponding to 284.8 eV (C─C/C═C), 286.2 eV (C─H), 286.9 eV (C─O), and 288.3 eV (C═O). The O 1s spectrum showed peaks at 531.4 eV (C═O), 532.7 eV (C─O), and 534.2 eV (adsorbed oxygen). This relatively high proportion of C─O suggests the formation of C─O─C bridge bonds through condensation during carbonization. Analysis of the N 1s spectrum revealed multiple nitrogen configurations, including pyridinic, pyrrolic, and graphitic N, indicating that nitrogen atoms were embedded within the carbon framework in a pyrrolic‐type configuration.

For Met‐CD (Figure S4, Supporting Information), the S 2p spectrum exhibited peaks at 163.6 and 164.8 eV attributed to the S 2p_3_/_2_ and S 2p_1_/_2_ components of a typical thioether structure (Figure 1J). The C 1s spectrum presented peaks at 284.8 eV (C─C/C═C), 286.5 eV (C─N/C─O), and 288.2 eV (C═O), consistent with those observed for Se‐Met‐CD. This confirms that sulfur from the methionine precursor was covalently integrated into the carbon framework without undergoing oxidation.

For Se‐Met‐CD (Figure S5, Supporting Information), the high‐resolution Se 3d spectrum displayed a doublet peak at 54.2 eV, characteristic of Se─C bonds (Figure 1K). No signals were detected in the 58–60 eV range, ruling out the presence of oxidized selenium species and confirming stable incorporation of Se in an organic‐bound state. Deconvolution of the C 1s spectrum revealed peaks at 284.8 eV (C─C/C═C), 286.1 eV (C─N/C─O/C─Se), and 288.3 eV (C = O), indicating a partially graphitized carbon core that retains amino and carboxylic groups. The N 1s spectrum exhibited a doublet at 399.8 and 401.1 eV, corresponding to neutral amine (−NH_2_) and protonated amine (−NH_3_^+^) functionalities, respectively. Collectively, these XPS results demonstrate that their amino acid precursors directly govern the chemical structures of CDs.

**Figure 2:**
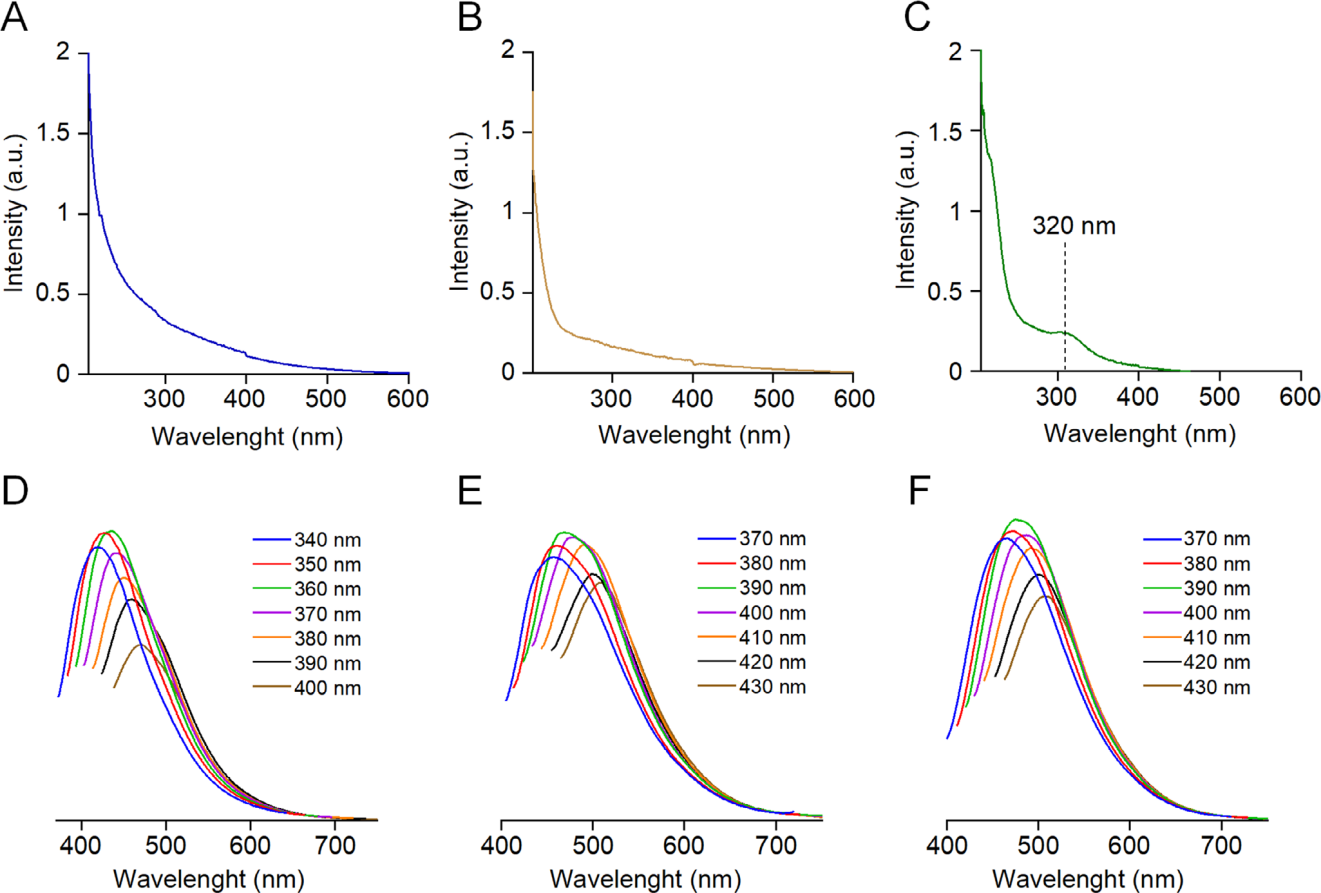
Optical properties of Val‐CD, Met‐CD, and Se‐Met‐CD. A─C) UV−Vis absorption spectra of Val‐CD A), Met‐CD B), and Se‐Met‐CD C) in aqueous solution. D─F) Photoluminescence spectra of Val‐CD D), Met‐CD E), and Se‐Met‐CD F).

The optical properties of CDs, which are critical for their biomedical applications, were subsequently characterized using ultraviolet–visible (UV–vis) absorption spectroscopy and photoluminescence (PL) measurements. No distinct absorption peaks were observed in the UV–vis spectra for Val‐CD (**Figure** **2**A) and Met‐CD (Figure 2B). In contrast, the UV–vis spectrum for the aqueous solution of Se‐Met‐CD displayed a pronounced absorption peak at 320 nm, which could be attributed to n→π* (carboxyl and/or C─N/or C─Se) transitions involving surface or molecular states (Figure 2C). This peak is slightly blueshifted compared to that of previously reported CDs derived from selenocystine, which exhibit a similar absorption maximum at ≈340 nm.^[^ ^23^ ^]^ This difference in peak position can be attributed to the inherent structural differences between the two precursors, as well as variations in the synthesis conditions.

Excitation‐dependent photoluminescence, a hallmark property of CDs, was also examined. Similar to other CDs, all three types of CDs exhibited typical excitation‐dependent photoluminescence, with fluorescence emission spectra shifted in response to different excitation wavelengths (Figure 2D–F). For example, altering the excitation wavelength for Val‐CD from 300 to 460 nm resulted in a red shift in emission from 415 to 525 nm. This is a common optical phenomenon observed in CDs, which further confirms the successful synthesis of all three types of CDs.^[^ ^25^ ^]^ The maximum PL intensity for Val‐CD was observed under excitation at 360 nm, whereas Met‐CD and Se‐Met‐CD both exhibited maximum PL intensity at 390 nm. To precisely characterize their optical parameters, excitation and emission spectra were acquired for each CD (Figure S6, Supporting Information). Val‐CD displayed a distinct excitation peak at 360 nm and a corresponding emission peak at 436 nm. In contrast, both Met‐CD and Se‐Met‐CD exhibited optimal excitation at 390 nm, with emission maxima centered at 470 nm. The close similarity in the optical behaviors of Met‐CD and Se‐Met‐CD is likely due to the structural resemblance of their precursor amino acids.

Given the core objective of developing a therapeutic strategy to alleviate oxidative stress, a comprehensive evaluation of the antioxidant capabilities of CDs was essential. A series of assays was conducted to compare the ROS‐scavenging performance of Se‐Met‐CD, Met‐CD, and Val‐CD.

**Figure 3:**
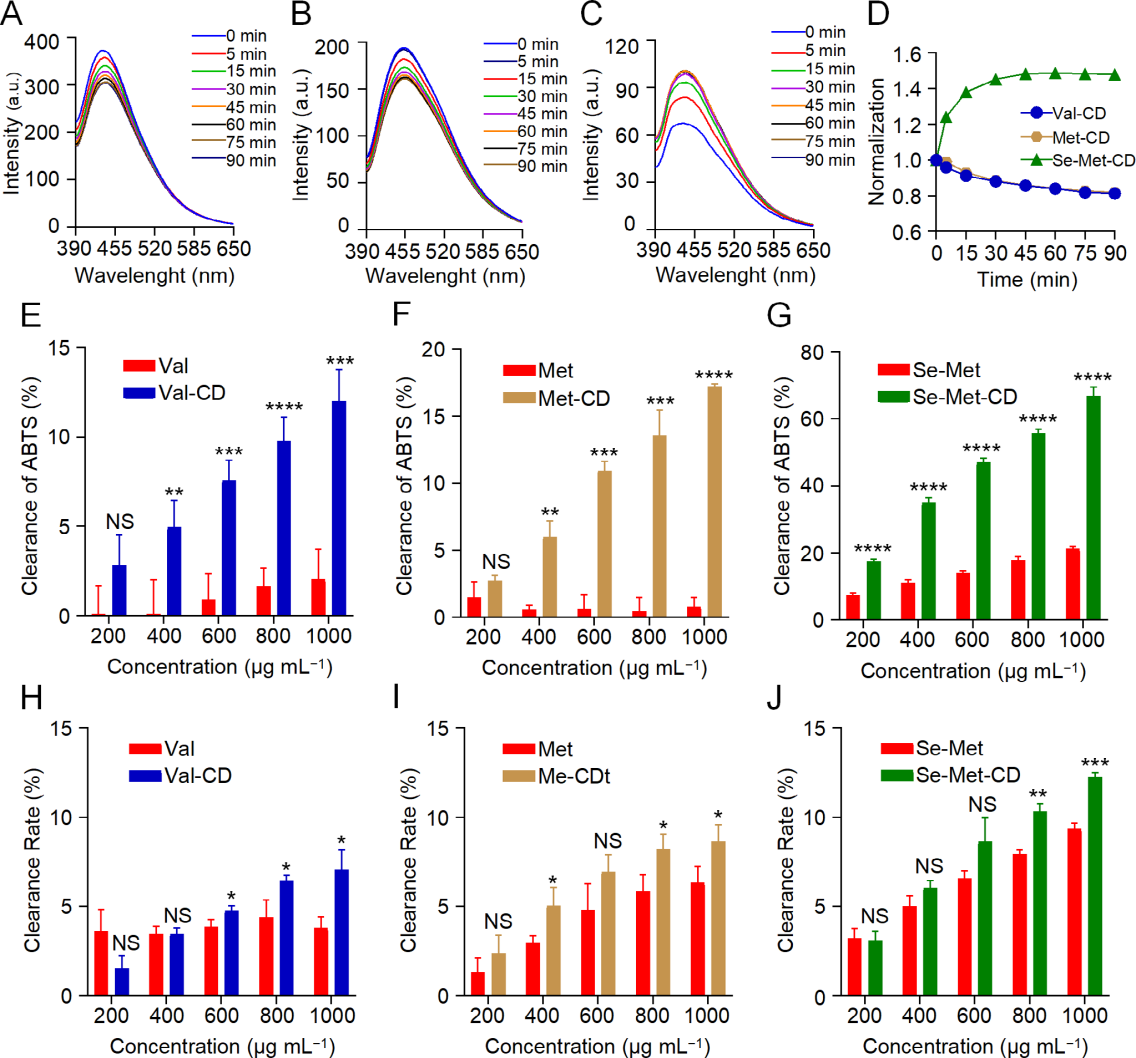
Antioxidant properties of Val‐CD, Met‐CD, and Se‐Met‐CD. A─C) Photoluminescence spectra of Val‐CD A), Met‐CD B), and Se‐Met‐CD C) after addition of 1.0 mm H_2_O_2_. D) Normalized fluorescence intensity at the peak emission wavelength forCDs shown in (A─C). E─G) Total antioxidant capacity of CDs and their precursor amino acids, as measured by ABTS assay. H─J) Scavenging ability of CDs and their precursor amino acids against DPPH radical. Val‐CD is represented in blue, Met‐CD in brown, and Se‐Met‐CD in green. All statistical comparisons shown are made between the performance of CDs and that of their respective precursor amino acids at each corresponding concentration. All statistical data are represented as mean ± SD (*n* = 3; NS, no significant difference; ^*^ *p* < 0.05, ^**^ *p* < 0.01, ^***^ *p* < 0.001, ^****^ *p* < 0.0001).

The initial assessment involved monitoring changes in the fluorescence spectra of CDs following the addition of hydrogen peroxide (H_2_O_2_) (**Figure** **3**A–C), with the normalized fluorescence intensity at the peak subsequently quantified (Figure 3D). Val‐CD (Figure 3A) and Met‐CD (Figure 3B) exhibited progressive fluorescence quenching upon the addition of H_2_O_2_, likely due to the oxidation of surface groups, such as hydroxyl and amino groups, which altered the chemical structure and disrupted the emissive states. In contrast, Se‐Met‐CD exhibited a gradual enhancement of fluorescence upon H_2_O_2_ addition (Figure 3C). This increase resulted from the oxidation of surface‐bound Se atoms. Initially, Se existed in a reduced state, acting as a nonradiative trap that quenched fluorescence. Oxidation of these groups to higher‐valent forms may have passivated surface defects and reduced nonradiative recombination, thereby enhancing the quantum yield. These results are consistent with previous reports on other Se‐containing CDs exposed to H_2_O_2_,^[^ ^23^ ^]^ and highlight the specificity of Se‐Met‐CD under oxidative conditions.

To quantify antioxidant potential, the total antioxidant capacity of CDs and their corresponding precursor amino acids was measured using the well‐established 2,2′‐azino‐bis(3‐ethylbenzothiazoline‐6‐sulfonic acid) (ABTS) assay (Figure 3E–G). All three CDs exhibited significantly higher antioxidant capacity compared to their precursor amino acids. Notably, Se‐Met‐CD exhibited an antioxidant capacity 3.1 times that of its Se‐Met precursor. Furthermore, its potency was ≈3.9 times that of Met‐CD and 5.6 times that of Val‐CD, respectively (all *p* < 0.0001). Among the precursors, only Se‐Met possessed detectable activity, while Met and Val showed negligible effects.

To validate these findings, the 2,2‐diphenyl‐1‐picrylhydrazyl (DPPH) radical scavenging ability of CDs and their precursors was also assessed (Figure 3H–J). Again, Se‐Met‐CD displayed the strongest DPPH scavenging ability, followed by Met‐CD and Val‐CD. However, the differences among the three CDs were less pronounced than in the ABTS assay, possibly owing to the different underlying reaction mechanisms.

In summary, Se‐Met‐CD exhibited the most potent antioxidant capacity, with Met‐CD showing moderate capacity and Val‐CD the weakest antioxidant activity. Critically, all three CDs outperformed their respective precursor amino acids. The enhanced fluorescence response of Se‐Met‐CD in the presence of H_2_O_2_, along with its favorable performances in both ABTS and DPPH assays, highlighted that the redox chemistry of Se was key to its efficient ROS‐scavenging activity. These findings underscore that Se‐doped CD functioned as more effective antioxidants than their undoped counterparts.

The superior antioxidant performance of Se‐Met‐CD can be attributed to the intrinsic redox properties of incorporated selenium atom. During synthesis, selenium from the Se‐Met precursor is doped into the carbon framework, creating highly reactive site. The sites likely exist in reduced forms, such as selenoether moieties (─Se─CH_3_), on the surface of CD. Selenium moieties serve to directly and efficiently neutralize ROS, such as H_2_O_2_, through an oxidation‐reduction reaction in which the selenium itself is oxidized (Scheme 1).^[^ ^26^ ^]^ This proposed mechanism is strongly supported by the fluorescence enhancement observed upon the addition of H_2_O_2_ (Figure 3A–C). The reduced selenium species act as non‐radiative traps that quench fluorescence, and their oxidation to species like selenoxide passivates these traps, leading to an increase in fluorescence intensity.^[^ ^23^ ^]^ In contrast, the sulfur atom in Met‐CD is less effective in this role. Due to the lower bond energy of C─Se compared to C─S and the higher nucleophilicity of selenols compared to thiols, the selenium active sites in Se‐Met‐CD possess a more favorable redox potential for ROS scavenging.^[^ ^27^ ^]^ This intrinsic chemical advantage explains why Se‐doping confers a substantially greater antioxidant capacity to CD than S‐doping, providing a clear rationale for developing selenium‐based materials to treat oxidative stress‐related diseases, such as IVDD.

### 2.2 Protection Efficacy and Mechanisms of Nucleus Pulposus Cells Through Suppressing Oxidative Stress by Se‐Met‐CD in vitro

Before evaluating the therapeutic potential, the biocompatibility of synthesized CDs was assessed using a Cell Counting Kit‐8 (CCK‐8) assay with NPCs (Figure S7, Supporting Information). After 24 h of incubation with CDs at concentrations ranging from 0.0 to 100.0 µg mL^−1^, no significant reduction in cell viability was observed, and survival rates remained statistically comparable to those of the Control group (*p* > 0.05). This result confirmed the biosafety of the three types of CDs. The cytocompatibility of precursor amino acids was also evaluated (Figure S8, Supporting Information).

**Figure 4:**
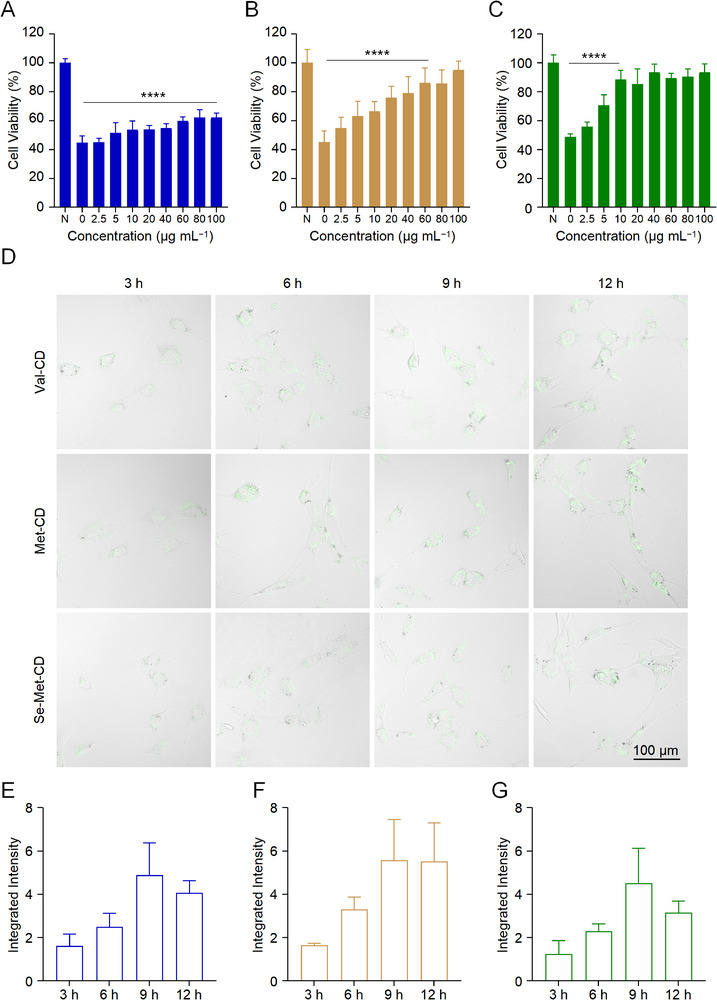
Cytoprotective effects and cell uptake of different CDs in NPCs in vitro. A─C) Viability of NPCs after 24 h of co‐incubation with various concentrations of Val‐CD A), Met‐CD B), and Se‐Met‐CD C) in the presence of 200 µm H_2_O_2_. D) Representative fluorescence microscopy images showing the uptake of CDs (100.0 µg mL^−1^) by NPCs. The images shown are composites created by overlaying the fluorescence and bright‐field channels. E─G) Quantitative analysis of integrated intensity of Val‐CD E), Met‐CD F), and Se‐Met‐CD G) using ImageJ software. Val‐CD is represented in blue, Met‐CD in brown, and Se‐Met‐CD in green. All statistical data are represented as mean ± SD (Figure A─C, *n* = 6; Figure E─G, *n* = 4; ^*^ *p *< 0.05, ^**^ *p* < 0.01, ^***^ *p* < 0.001, ^****^ *p* < 0.0001).

To establish an oxidative stress model, NPCs were subsequently treated with different concentrations of H_2_O_2_ for 24 h. A concentration of 200.0 µm H_2_O_2_ was selected for subsequent experiments, as it reduced cell viability to ≈50% (Figure S9, Supporting Information). NPCs were then co‐incubated with 200.0 µm H_2_O_2_ and varying concentrations of each CD for 24 h, and cell viability was measured (**Figure** **4**A─C). Se‐Met‐CD exhibited the most pronounced protective effect, with 10.0 µg mL^−1^ restoring cell viability to 88.6% of the control level. Higher concentrations did not yield additional benefits. For Met‐CD, cell viability reached ≈86.3% at a concentration of 60.0 µg mL^−1^. Val‐CD at 100.0 µg mL^−1^ produced only a slight improvement in viability and was significantly less effective than Se‐Met‐CD and Met‐CD.

Cells were also co‐incubated with H_2_O_2_ and the precursor amino acids. Se‐Met at 80.0 µg mL^−1^ restored viability to 88.9% of the Control group (Figure S10, Supporting Information), and higher concentrations provided no further improvement. Met demonstrated antioxidant activity only at a high concentration of 400.0 µg mL^−1^, while Val showed no protective effect. Thus, all three types of CDs exhibited improved antioxidant performance compared to their respective precursor amino acids. Based on these findings, 10.0 µg mL^−1^ for Se‐Met‐CD and 60.0 µg mL^−1^ for Met‐CD were selected as the optimal protective concentrations. Val‐CD at a concentration of 100.0 µg mL^−1^ was used as a reference group in subsequent experiments.

To evaluate cell uptake and probe utility, the intracellular localization and fluorescent behaviors of CDs were investigated. NPCs were co‐cultured with 100.0 µg mL^−1^ of each CD, and microscopy confirmed successful uptake, as indicated by strong and stable intracellular fluorescence signals (Figure 4D). ImageJ was used to quantify the integrated fluorescence intensity from confocal images (Figure 4E–G). The results indicated that all three types of CDs were efficiently internalized by the cells within 3 h, with intracellular fluorescence intensity peaking at 9 h. CDs were predominantly localized in the cytoplasm, consistent with previous findings.^[^ ^28^ ^]^ After 12 h, fluorescence intensity declined slightly. Weaker fluorescence was observed for Se‐Met‐CD, which was attributed to its lower quantum yield. The average fluorescence intensity of Met‐CD was similar to that of Val‐CD, likely because the 488 nm laser line used for excitation more closely matched the optimal excitation range of Met‐CD. Complete imaging data are presented in Supplementary Figure S11. Our findings clearly demonstrate the efficient internalization of synthesized CDs within NPCs. While the precise molecular machinery governing this uptake was not experimentally determined, understanding the pathway is critical for interpreting the observed biological activity. The literature describes several potential endocytic pathways for nanoparticles of this size, including clathrin‐mediated endocytosis, caveolae‐mediated endocytosis, and micropinocytosis.^[^ ^29^ ^]^ Future mechanistic studies employing specific endocytic inhibitors will be necessary to elucidate this crucial mechanism definitively. To visually confirm the protective effects of CDs against oxidative‐stress‐induced cell death, calcein‐Acetoxymethyl ester (AM)/propidium iodide (PI) live/dead staining was performed (Figure S12, Supporting Information). NPCs were treated with DMEM/F12 containing either Se‐Met‐CD (10.0 µg mL^−1^), Met‐CD (60.0 µg mL^−1^), or Val‐CD (100.0 µg mL^−1^), and co‐exposed to H_2_O_2_ at a final concentration of 300.0 µm. After 8 h of incubation, the treatment medium was removed, and cell death rates were quantified. Treatment with Se‐Met‐CD at a concentration of 10.0 µg mL^−1^ demonstrated the most potent protective effect, markedly reducing the cell death rate to only 5.6% compared to 18.8% for the H_2_O_2_‐treated Control group. Met‐CD at 60.0 µg mL^−1^ also provided substantial protection, resulting in a death rate of 8.3%. In comparison, the effect of Val‐CD was less pronounced, with a death rate of 15.1%. Corroborating the CCK‐8 viability results, these findings underscore the robust cytoprotective role of Se‐Met‐CD against oxidative stress.

**Figure 5:**
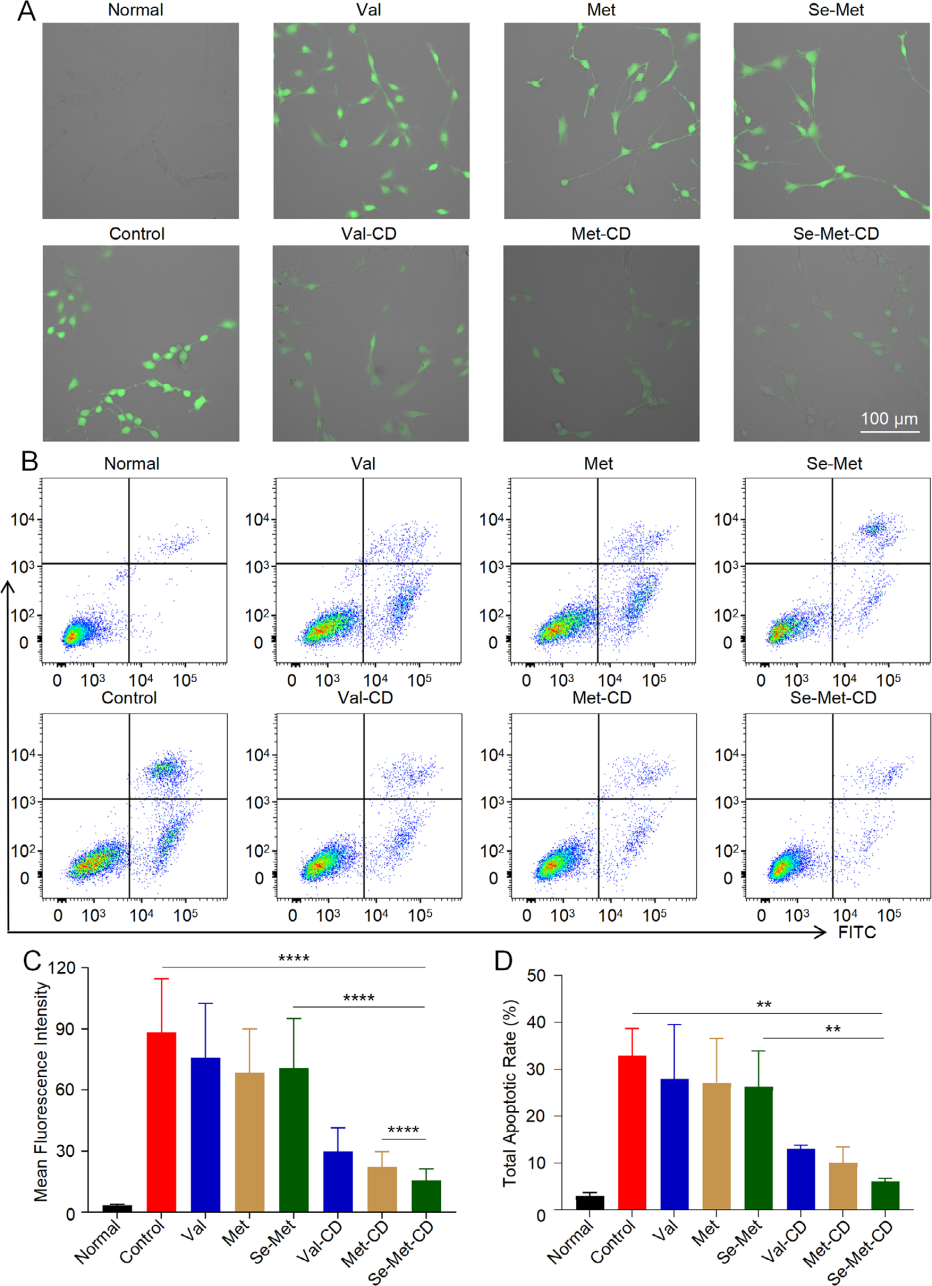
Intracellular ROS scavenging and anti‐apoptotic effects of CDs in NPCs under oxidative stress in vitro. A) Fluorescence microscopy images of intracellular ROS levels in NPCs, detected using a fluorescent probe after co‐incubation with H_2_O_2_ and respective CDs. The images are overlays of the fluorescence and bright‐field channels. B) Flow cytometry analysis of apoptosis in NPCs after co‐incubation with H_2_O_2_ and respective CDs, using an Annexin V‐FITC/PI double staining assay. C) Quantitative analysis of ROS levels from (A) using ImageJ software. D) Statistical analysis of total apoptosis rate from flow cytometry data shown in (B). Val‐CD is represented in blue, Met‐CD in brown, and Se‐Met‐CD in green. All statistical data are represented as mean ± SD (*n* = 3; NS, no significant difference; ^*^ *p* < 0.05, ^**^ *p* < 0.01, ^***^ *p* < 0.001, ^****^ *p* < 0.0001).

To determine whether the observed cytoprotective effects were attributable to the scavenging of ROS, intracellular ROS levels in NPCs were measured. To analyze intracellular ROS levels, NPCs were co‐incubated with H_2_O_2_ and the respective compounds and were subsequently visualized using an intracellular ROS detection probe and confocal microscopy (**Figure** **5**A). The images in Figure 5 are overlays of the bright‐field and fluorescence channels, where green fluorescence indicates the intracellular ROS levels. As shown, ROS levels increased significantly following stimulation with H_2_O_2_ (Figure 5C). Treatment with 100.0 µg mL^−1^ Val‐CD, 60.0 µg mL^−1^ Met‐CD, and 10.0 µg mL^−1^ Se‐Met‐CD reduced ROS levels to 33.8%, 25.1%, and 17.7%, respectively, relative to the H_2_O_2_‐only group. In contrast, treatment with the three precursor amino acids did not result in any significant reduction in ROS levels. These findings confirm that the amino‐acid‐derived CDs, particularly Se‐Met‐CD, effectively scavenge intracellular ROS under conditions of oxidative stress.

Excessive oxidative stress is known to cause apoptosis in NPCs. Therefore, the rate of apoptosis was used as a key indicator of oxidative damage. Apoptosis was evaluated using Annexin V‐fluorescein isothiocyanate (FITC)/PI double staining and flow cytometry. The results are plotted in Figure 5B, where the x‐axis represents the fluorescence intensity of FITC and the y‐axis represents the fluorescence intensity of PI, respectively. The Q4 quadrant in these plots corresponds to early apoptotic cells, while the Q1 quadrant represents late apoptotic or necrotic cells. All three CDs significantly reduced both early and late apoptosis compared to the H_2_O_2_‐treated Control group.

The total percentages of apoptotic cells were calculated to quantify the protective effects of CDs (Figure 5D). Treatment with 10.0 µg mL^−1^ of Se‐Met‐CD offered the most significant protection, reducing the total apoptosis rate from 32.9% in the H_2_O_2_‐treated Control group to just 6.1%. Treatment with 60.0 µg mL^−1^ of Met‐CD also effectively mitigated apoptosis, resulting in a rate of 10.0%, while 100.0 µg mL^−1^ of Val‐CD showed a less pronounced effect, with a rate of 13.0%. In contrast, the anti‐apoptotic effects of precursor amino acids were substantially weaker than those observed for their corresponding CDs.

In summary, Se‐Met‐CD and Met‐CD effectively protected NPCs from oxidative‐stress‐induced damage by scavenging intracellular ROS and inhibiting both early and late apoptosis under oxidative stress conditions in vitro. These CDs exhibited stronger antioxidant capabilities than their precursor amino acids.

**Figure 6:**
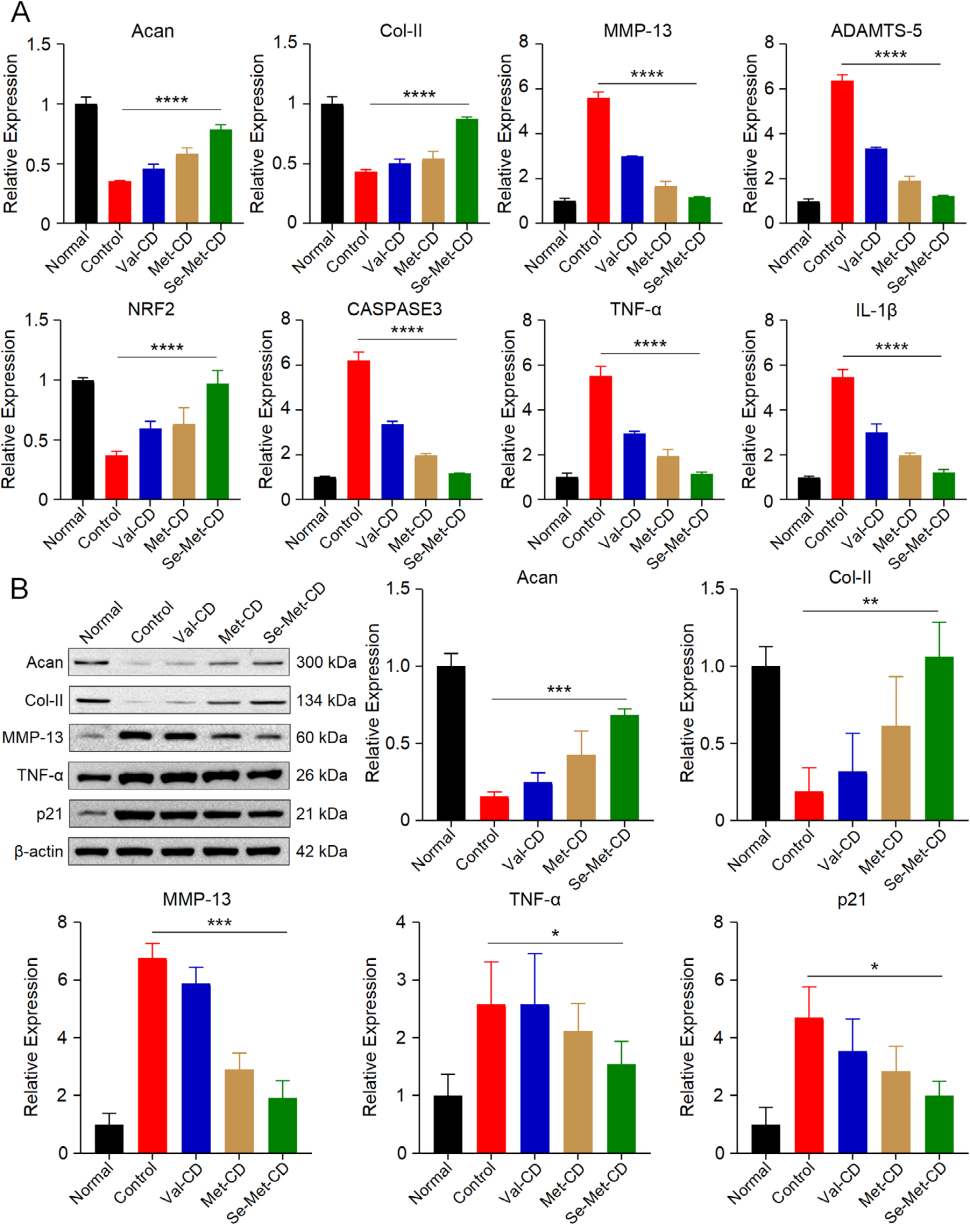
Mechanistic investigation of how Val‐CD, Met‐CD, and Se‐Met‐CD prevent oxidative‐stress‐induced cell death in vitro. A) Relative mRNA expression levels in NPCs from each group, as determined by quantitative PCR. Genes analyzed include those related to matrix synthesis (Acan and Col‐II), matrix degradation (MMP‐13 and ADAMTS‐5), antioxidant defense (Nrf2), apoptosis (caspase‐3), and inflammation (TNF‐α and IL‐1β). B) Western blot analysis and corresponding semi‐quantitative analysis of protein expression levels in NPCs from each group. Proteins analyzed include Acan, Col‐II, MMP‐13, TNF‐α, and cellular senescence‐related protein p21. Val‐CD is represented in blue, Met‐CD in brown, and Se‐Met‐CD in green. The corresponding uncropped original blots are presented in Figures S13–S17 (Supporting Information). All statistical data are represented as mean ± SD (*n* = 3; ^*^ *p *< 0.05, ^**^ *p *< 0.01, ^***^ *p *< 0.001, ^****^ *p *< 0.0001).

To elucidate the molecular mechanisms underlying the anti‐apoptotic and antioxidant effects of CDs, changes in gene and protein expression were analyzed using quantitative PCR (**Figure** **6**A) and Western blotting (Figure 6B), respectively.

At the transcriptional level, the expression of aggrecan (Acan) and collagen type II (Col‐II), two key genes involved in cartilage matrix synthesis, was examined. Acan serves as a core component of ECMs and a marker of matrix anabolism, while Col‐II forms a fibrous network that maintains disc structure and mechanical function.^[^ ^30^ ^]^ In the H_2_O_2_‐treated Control group, the expression of both Acan and Col‐II was markedly suppressed to 35.3% and 43.3%, respectively, relative to the Normal group (normalized to 1), indicating impaired ECM synthesis. While Val‐CD and Met‐CD moderately alleviated this suppression, Se‐Met‐CD was substantially more effective, restoring the expression of Acan and Col‐II to 78.3% and 87.3%, respectively, nearing the levels of the untreated Normal group.

The effects of CDs on matrix catabolism were evaluated by analyzing the expression of matrix‐degrading enzymes. A disintegrin and metalloproteinase with thrombospondin motifs 5 (ADAMTS‐5) is the primary enzyme responsible for degrading Acan, while matrix metalloproteinase 13 (MMP‐13) degrades ECM components, such as Col‐II and collagen IX. Up‐regulation of both enzymes is closely associated with ECM degradation in IVDD.^[^ ^15^ ^]^ In the Control group, the expression levels of MMP‐13 and ADAMTS‐5 were 5.6 and 6.4 times those of the Normal group. Treatment with Val‐CD reduced these levels to 3.0 and 3.3 times that of the Normal group. Met‐CD further suppressed expression to 1.7 and 1.9 times that of the Normal group, while Se‐Met‐CD was the most potent, lowering expression to just 1.2 and 1.2 times that of the Normal group. Crucially, this potent suppression by Se‐Met‐CD resulted in a reduction of MMP‐13 and ADAMTS‐5 expression to 20.9% and 19.1% of the levels observed in the H2O2‐treated Control group, respectively.

Next, the expression of genes involved in antioxidant defense and apoptosis was investigated. Nuclear factor erythroid 2‐related factor 2 (Nrf2) is a key transcription factor that regulates the expression of antioxidant genes, including heme oxygenase‐1 and NAD(P)H quinone oxidoreductase 1, and plays a central role in maintaining redox homeostasis.^[^ ^31^ ^]^ Se‐Met‐CD significantly up‐regulated the expression of Nrf2 in NPCs, restoring it to 97.0% of normal levels, a substantial increase from the 37.0% observed in the H_2_O_2_‐treated Control group. This up‐regulation activates the cellular antioxidant defense system, thereby mitigating damage from oxidative stress. Caspase‐3 is a key effector protease with a central role in the apoptotic process.^[^ ^32^ ^]^ In control NPCs subjected to oxidative stress, the expression level of caspase‐3 was up‐regulated 6.2‐fold, leading to a marked increase in apoptosis. Se‐Met‐CD reduced caspase‐3 expression to 18.7% of control levels, confirming its ability to alleviate oxidative‐stress‐induced apoptosis.

To complete the genetic analysis, the expression of pro‐inflammatory cytokines was evaluated. Tumor necrosis factor‐α (TNF‐α) is a central pro‐inflammatory mediator that amplifies inflammation and contributes to cell damage and apoptosis.^[^ ^33^ ^]^ Interleukin‐1β (IL‐1β) promotes the expression of ECMs‐degrading enzymes, such as MMP‐13, and plays a critical role in diseases like IVDD and osteoarthritis.^[^ ^34^ ^]^ Following oxidative stress, TNF‐α and IL‐1β levels were up‐regulated to both 5.5‐fold. The expression of both cytokines was significantly down‐regulated in the Se‐Met‐CD and Met‐CD groups, with the Se‐Met‐CD group exhibiting the most potent effect, suppressing TNF‐α and IL‐1β expression levels to 20.8% and 22.1% of their respective control levels.

To validate the genetic expression results at the protein level, Western blotting was conducted (Figure 6B). The complete uncropped blots for this analysis are available in Supplementary Figures S13–S17. Oxidative stress markedly reduced protein levels of Acan and Col‐II in NPCs to just 15.6% and 18.9% of normal levels, respectively. In the Se‐Met‐CD group, however, the expression of both proteins was substantially restored, with Acan reaching 68.4% and Col‐II reaching 106.2% of normal levels. Compared to the Normal group, MMP‐13 protein levels in control NPCs increased 6.8‐fold, indicating accelerated matrix degradation. Se‐Met‐CD markedly suppressed this up‐regulation, reducing MMP‐13 expression to a level 1.91 times that of the Normal group, thereby demonstrating its ability to help preserve ECM integrity. TNF‐α protein levels remained high in the Val‐CD group and were only slightly reduced by Met‐CD. In contrast, Se‐Met‐CD significantly down‐regulated TNF‐α expression to 62.6% of the control level, indicating a strong anti‐inflammatory effect. Furthermore, oxidative stress induced a 4.7‐fold up‐regulation in the expression of cellular senescence marker cyclin‐dependent kinase inhibitor 1 (p21). Se‐Met‐CD effectively counteracted this effect, suppressing p21 expression to a level just 2.0 times that of the Normal group. This result indicates that Se‐Met‐CD inhibits oxidative stress‐induced cellular senescence.

Collectively, these results indicate that the superior protective efficacy of Se‐Met‐CD against oxidative stress stems from its potent intrinsic antioxidant properties, which enable it to scavenge intracellular ROS, thereby mitigating oxidative stress effectively. This critical upstream intervention effectively disrupts the cascade of downstream damage triggered by oxidative stress. It restores ECM homeostasis via a dual‐regulatory mechanism, simultaneously up‐regulating key anabolic genes, Acan and Col‐II, while potently suppressing core matrix‐degrading enzymes, MMP‐13 and ADAMTS‐5. Se‐Met‐CD also activates the endogenous Nrf2 antioxidant pathway, thereby bolstering intrinsic cellular defense capabilities. Furthermore, it suppresses the expression of pro‐inflammatory cytokines, TNF‐α and IL‐1β, and down‐regulates key mediators of apoptosis, such as caspase‐3 and senescence marker p21, thus preserving the viability and function of NPCs. Crucially, all of these multifaceted protective effects were substantially more pronounced for Se‐Met‐CD compared to its counterparts, Met‐CD and Val‐CD.

In summary, these findings systematically reveal the multifaceted mechanism by which Se‐Met‐CD modulates ECM metabolism, inflammation, apoptosis, and senescence by alleviating oxidative stress. This comprehensive evidence supports the application of Se‐Met‐CD as a promising nano‐interventional strategy for IVDD.

### 2.3 Attenuation Efficacy of Intervertebral Disc Degeneration by Se‐Met‐CD in vivo

Having established the protective effects of Se‐Met‐CD and Met‐CD against oxidative stress in vitro, their therapeutic efficacy was next validated in vivo using a rat model of IVDD. Se‐Met‐CD at 10.0 µg mL^−1^ and Met‐CD at 60.0 µg mL^−1^ were selected for the animal experiments, based on their optimal performance in vitro. A previously reported thermo‐sensitive hydrogel, poly(amino acid) methoxy poly(ethylene glycol)‐*block*‐poly(L‐methionine) (mPEG‐*b*‐PMet, PM) was used as a delivery vehicle for intradiscal injection.^[^ ^35^ ^]^

Spectroscopic analyses confirmed the successful synthesis of the PM copolymer. In the ^1^H NMR spectrum (Figure S18A, Supporting Information), characteristic proton signals for the methionine segment appeared at 4.8, 2.6, and 2.1 ppm. The degree of polymerization of PMet block was determined to be 25 by comparing the integral of its methine proton signal (4.8 ppm) with that of the repeating ethylene units in the mPEG block (3.8 ppm). Further structural validation was provided by FT‐IR analysis, which identified key functional groups including secondary amines (3295.0 cm^−1^), carbonyls (1657.5 cm^−1^), ethers (1306.0 cm^−1^), and thioethers (1107.0 cm^−1^) (Figure S18B, Supporting Information).

The PM copolymer demonstrated excellent thermo‐sensitivity, with its aqueous solutions transitioning from a liquid‐like sol to a solid‐like gel upon heating. This behavior was characterized by a concentration‐dependent critical gelation temperature (CGT), which was 32 °C at 5.0 wt.% and decreased to 18 °C at 8.0 wt.% (Figure S18C, Supporting Information). The CGT values below physiological temperature ensure rapid gelation after injection. Rheological measurements supported this observation, showing a dramatic surge in the storage modulus (G') from 3.4 Pa (sol state at 20 °C) to 229.0 Pa (gel state at 37 °C), signifying the formation of a robust gel (Figure S19, Supporting Information). Morphologically, SEM imaging revealed that the hydrogel possesses a highly ordered and interconnected internal network structure (Figure S20, Supporting Information).

To further investigate the in vivo fate of the nanoparticles and address the crucial question of biodistribution, we conducted a preliminary study using Cy5.5‐labeled Se‐Met‐CDs. Following a single intradiscal injection, ex vivo fluorescence imaging of major organs and the injection site was performed after 24 h (Figure S21, Supporting Information). The results revealed a strong and distinct fluorescence signal that was clearly localized to the injected caudal vertebral region. In stark contrast, no detectable fluorescence was observed in the harvested heart, liver, spleen, lungs, or kidneys. This provides direct evidence that the Se‐Met‐CDs are well‐retained at the target site with minimal leakage into the systemic circulation, a finding consistent with both the small injection dose and the enclosed, avascular nature of the intervertebral disc.

**Figure 7:**
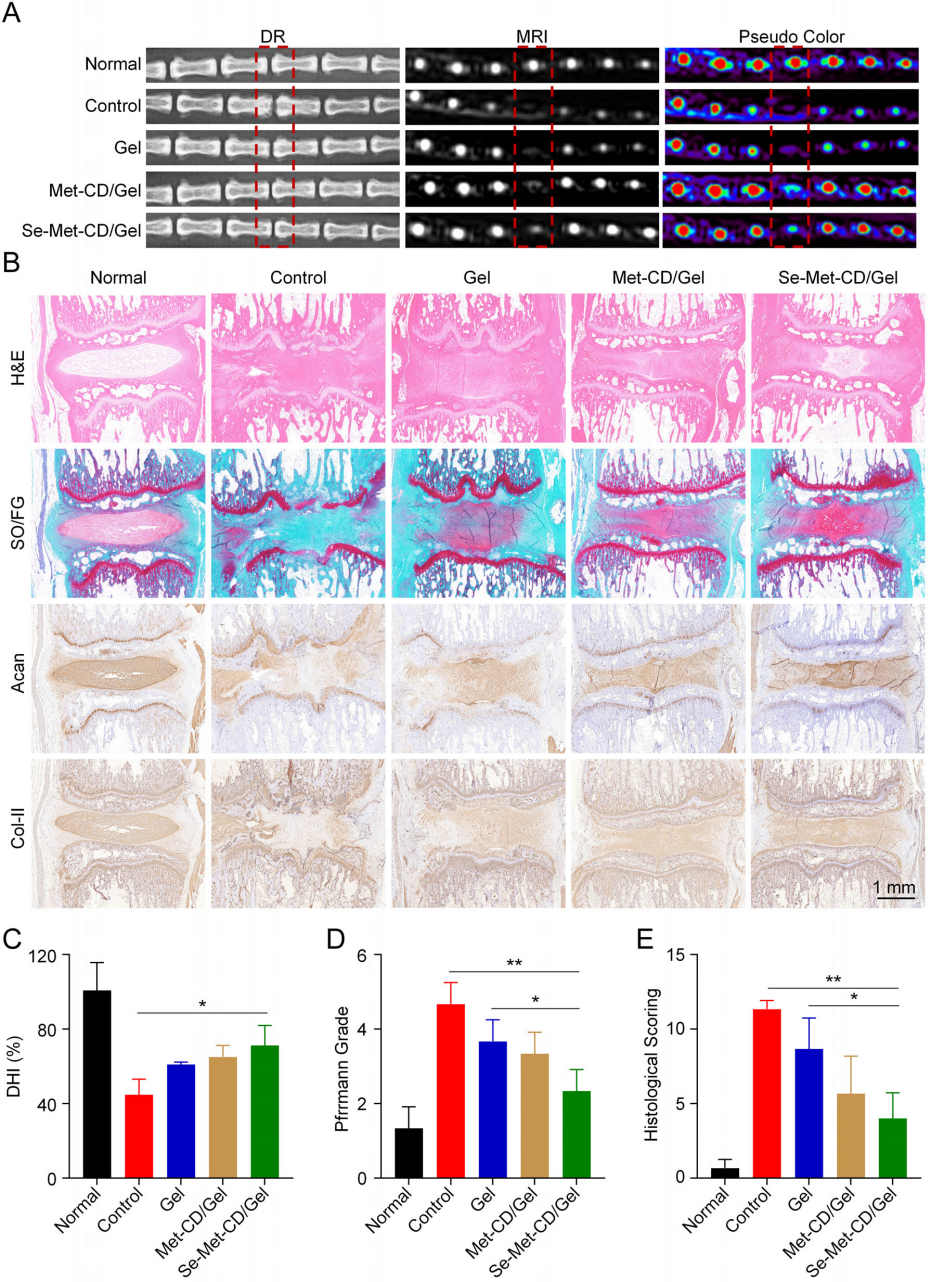
Radiographic and histological evaluation of animal tissues at eight weeks post‐operation. A) Representative X‐ray and MRI images of rat caudal vertebrae showing the Co7/8 (operated) segments. Pseudocolor was applied to the MRI scans to enhance visualization. B) Histological and immunohistochemical staining of disc sections from the Co7/8 level. From top to bottom: H&E staining, Safranin O/Fast Green staining, and immunohistochemistry for Acan and Col‐II. C) Quantification of DHI as a percentage for each group. D) Pfirrmann scores for each group. E) Histological scores for each group. The red boxes indicate the Co7/8 disc space. All statistical data are represented as mean ± SD (*n* = 3; ^*^ *p *< 0.05, ^**^ *p *< 0.01).

IVDD was induced at the intervertebral disc between the 7^th^ and 8^th^ caudal vertebrae (Co7/8) via needle puncture as detailed in the Experiment Section. To generate a more severe model and rigorously assess therapeutic outcomes, the degree of needle rotation was increased to create a more challenging pathological environment. Evaluations were performed at 4 and 8 weeks post‐surgery. At each time point, rats underwent X‐ray and magnetic resonance imaging (MRI) scanning (**Figure** **7**A), followed by euthanasia and harvesting of caudal intervertebral discs for histological examination (Figure 7B).

At the 4‐ and 8‐week endpoints, disc height was first assessed via Digital Radiography (DR). At four weeks, the Control group showed a degree of narrowing of the intervertebral disc space compared to the Normal group, while the Gel, Met‐CD/Gel, and Se‐Met‐CD/Gel groups exhibited less severe narrowing (Figure S22, Supporting Information). Assessment of the disc height index (DHI) revealed that the DHI in the puncture‐induced Control group was 62.9% of that in the Normal group, whereas the Gel, Met‐CD/Gel, and Se‐Met‐CD/Gel groups retained DHIs of 68.7%, 67.7%, and 75.5%, respectively (Figure S23, Supporting Information).

By the 8‐week endpoint, the Control group exhibited a more pronounced narrowing of the intervertebral disc space and evidence of bone damage, in contrast to the Normal group (Figure 7A). A significant decrease in the DHI was observed to accompany this. The Gel group retained significantly more disc height than the Control group, and both the Se‐Met‐CD/Gel and Met‐CD/Gel groups exhibited substantially higher DHI values compared with those of the Control group. However, no statistically significant difference in DHI was observed among the Se‐Met‐CD/Gel, Met‐CD/Gel, and the Gel group (Figure 7C).

To assess internal disc health, a T2‐weighted MRI was used to evaluate hydration and degeneration of the nucleus pulposus (NP). At four weeks, normal discs exhibited a high‐intensity T2 signal, which was nearly absent in the IVDD‐induced Control group. Hydrogel treatment alone preserved a weak T2 signal, while Se‐Met‐CD/Gel treatment retained significantly more signal intensity (Figure S24, Supporting Information). Pseudocolor enhancement was applied to better visualize the differences among the groups. Quantitative grading using the Pfirrmann system revealed average scores of 4.3, 3.3, 2.7, and 2.3 for the Control, Gel, Met‐CD/Gel, and Se‐Met‐CD/Gel groups, respectively (Figure S25, Supporting Information). Similar conclusions were drawn at eight weeks (Figure 7A). At this time point, the average Pfirrmann scores were 4.7, 3.7, 3.3, and 2.3 for the Control, Gel, Met‐CD/Gel, and Se‐Met‐CD/Gel groups, respectively (Figure 7D). These results indicate significantly improved outcomes in the Se‐Met‐CD/Gel group.

Histological analysis was performed to further examine tissue morphology and ECM composition. Intervertebral discs were sectioned at 4 and 8 weeks post‐injection and stained with hematoxylin and eosin (H&E) and Safranin O/Fast Green staining (SOFG). At four weeks, Normal discs displayed a well‐organized structure with clear NP boundaries, while in Control discs, the NP tissue had almost completely disappeared (Figure S26, Supporting Information). The Gel group retained more proteoglycans, but its NP architecture remained disrupted. The Met‐CD/Gel group preserved disc height and partially restored NP structure, while the Se‐Met‐CD/Gel group yielded the most intact morphology with well‐defined NP boundaries. By eight weeks, Normal group discs displayed well‐organized structure with clear NP boundaries, while Control discs showed marked height loss and near‐complete absence of NP tissue (Figure 7B). The Gel group retained some disc height, but NP architecture remained disrupted. The Met‐CD/Gel group preserved disc height and partially restored NP structure, while the Se‐Met‐CD/Gel group yielded the most intact morphology with well‐defined NP boundaries.

Histological scoring was used to quantify the degree of degeneration. At four weeks, average scores were 10.3 for the Control group and 8.7, 5.3, and 3.0 for the Gel, Met‐CD/Gel, and Se‐Met‐CD/Gel groups, respectively (Figure S27, Supporting Information). By eight weeks, these scores increased to 11.3 for the Control group and 8.7, 5.7, and 4.0 for the Gel, Met‐CD/Gel, and Se‐Met‐CD/Gel groups, respectively, indicating progressive degeneration (Figure 7E). At both time points, both CD‐treated groups had significantly lower scores than the Control group, and Se‐Met‐CD showed the most significant mitigating effect on IVDD.

To further interrogate the anabolic state of ECMs at the protein level, immunohistochemical staining for Acan and Col‐II was performed on the intervertebral disc sections (Figure 7B). As anticipated, the Normal group displayed robust and extensive positive staining for both Acan and Col‐II within the nucleus pulposus, confirming a high baseline expression of these matrix proteins. In stark contrast, the Control group exhibited a marked reduction in both the area and intensity of Acan and Col‐II staining, indicative of severely compromised matrix synthesis. Although the hydrogel‐only treatment offered a slight degree of mitigation, the profound loss of both Acan and Col‐II persisted, indicating it was largely insufficient to halt matrix degradation. While the Met‐CD/Gel group achieved a partial restoration of Acan and Col‐II expression, a far more profound effect was observed in the Se‐Met‐CD/Gel group. Notably, the Se‐Met‐CD/Gel group demonstrated the most robust protective capacity, reestablishing the intensity and distribution of Acan and Col‐II staining to the greatest extent among all treatment groups. By potently up‐regulating the synthesis of its principal structural proteins, Se‐Met‐CD/Gel effectively preserves ECM homeostasis, thereby attenuating the overall progression of IVDD.

Together, the X‐ray, MRI, and histological data indicate that Se‐Met‐CD/Gel and Met‐CD/Gel groups conferred superior protection against IVDD compared with the Control group. Disc height preservation, NP hydration, and structural integrity were all significantly improved in the CD‐treated groups. Notably, the Se‐Met‐CD/Gel group demonstrated the most pronounced therapeutic effect. These in vivo outcomes are consistent with our in vitro findings and further support the enhanced antioxidative and protective capacity of Se‐Met‐CD over Met‐CD.

## 3 Conclusion

In this study, Se‐Met‐CD, Met‐CD, and Val‐CD were successfully synthesized, and the critical role of selenium in enhancing the therapeutic efficacy of CDs for alleviating IVDD was systematically demonstrated. A direct comparison among CDs derived from Se‐Met, Met, and Val confirmed that Se incorporation markedly improves the antioxidant properties. Se‐Met‐CD, with an average size of ≈3.08 nm, exhibited the strongest antioxidant capacity, 3.1 times that of Se‐Met and ≈3.9 times that of Met‐CD, as measured by ABTS assay. This enhanced redox activity was essential for protecting NPCs from oxidative damage. At a low concentration of 10.0 µg mL^−1^, Se‐Met‐CD restored cell viability to 88.6% under H_2_O_2_‐induced stress conditions, a protective effect comparable to that of Met‐CD at 60.0 µg mL^−1^, a concentration six times higher. Mechanistically, Se‐Met‐CD effectively scavenged intracellular ROS, reducing ROS levels to just 17.7% of those in the H_2_O_2_‐only group and decreasing the total apoptosis rate from 32.9% to 6.1%. This cytoprotective effect was achieved by restoring cellular homeostasis.

Se‐Met‐CD promoted a favorable matrix metabolism, evidenced by the up‐regulation of synthesis genes and significant suppression of matrix‐degrading enzymes and inflammatory cytokines. Specifically, it reduced the expression of MMP‐13 and ADAMTS‐5 to 20.9% and 19.1% of the levels found in the H_2_O_2_‐treated Control group, respectively. In a rat model of severe IVDD, intradiscal delivery of Se‐Met‐CD encapsulated in hydrogel significantly alleviated disc degeneration. This was reflected by an improved average Pfirrmann grade (2.3 vs. 4.7) and a superior histological score (4.0 vs. 11.3). In summary, these findings highlight the significance of Se in developing high‐performance antioxidant nanomaterials and establish Se‐Met‐CD as a promising therapeutic candidate for IVDD. By effectively scavenging ROS and thus neutralizing oxidative stress, rebalancing matrix metabolism, and mitigating inflammation and apoptosis, Se‐Met‐CD offers a compelling nano‐interventional strategy for disc degeneration treatment. Future investigations will focus on a systematic comparison between Se‐Met‐CD and other forms of selenium nanomaterials to further elucidate the structure‐dependent therapeutic mechanisms and optimize nano‐interventional strategies for IVDD.

## 4 Experimental Section

This section details the synthesis and antioxidant evaluation of CDs, the preparation of CD‐loaded hydrogels, the in vivo therapeutic evaluation in a rat model of IVDD, and statistical methods. The remaining Experimental Section is included in Supporting Information.

### Preparation of Se‐Met‐CD, Met‐CD, and Val‐CD

CDs were synthesized via one‐step pyrolysis using single amino acid precursors. Briefly, 1.0 g of the amino acid was ground into a fine powder using an agate mortar for 15 min. The powder was transferred to a crucible and heated in an oven at 160 °C (Se‐Met), 180 °C (Met), or 220 °C (Val), respectively. The resulting product was dissolved in 30.0 mL of deionized water and sonicated for 30 min. The solution was centrifuged at 12,000 rpm for 15 min to separate the soluble fraction from insoluble residues. The supernatant was then filtered through a 0.22 µm syringe filter. The filtrate was dialyzed against deionized water (molecular weight cut‐off 500 Da) for 24 h, with the water being changed every 2−4 h. The final product was obtained by freeze‐drying the purified solution.

### Evaluation of Antioxidant Capacity of Carbon Dots

To evaluate their direct H_2_O_2_ scavenging ability, 200.0 µg mL^−1^ CD solutions were mixed with a 2.0 mm H_2_O_2_ solution at a 1:1 volume ratio, and the resulting changes in fluorescence intensity were measured using a fluorescence spectrophotometer. In addition, total antioxidant capacity and free radical scavenging ability were quantified using an ABTS assay kit and a DPPH assay kit, respectively. Both assays were performed in strict accordance with the manufacturer's instructions.

### Measurement of Intracellular Reactive Oxygen Species Levels

Intracellular ROS levels were measured using a commercial ROS assay kit. NPCs were seeded in glass‐bottom dishes at a density of 40,000 cells per dish and incubated for 16 h. Subsequently, the cells were treated with DMEM/F12 medium containing either Se‐Met‐CD at a concentration of 10.0 µg mL^−1^, Met‐CD at 60.0 µg mL^−1^, Val‐CD at 100.0 µg mL^−1^, or their corresponding precursor amino acids at the same respective concentrations. All treatment groups were co‐exposed to H_2_O_2_ at a final concentration of 200.0 µm and incubated for 8 h. Following treatment, the cells were washed three times with phosphate–buffered saline (PBS) and incubated with the ROS detection probe for 30 min, as per the manufacturer's instructions. Intracellular fluorescence was visualized using a confocal laser scanning microscope, and the mean fluorescence intensity was quantified using ImageJ software (version 1.8.0, National Institutes of Health, Bethesda, MD, USA; https://imagej.net/).

### Preparation of Carbon Dot‐Loaded Hydrogels

In this study, a previously reported thermo‐sensitive poly(amino acid) hydrogel, methoxy poly(ethylene glycol)‐*block*‐poly(L‐methionine) (mPEG_45_‐*b*‐PMet_25_, PM), was selected as the in vivo delivery vehicle for CDs. This hydrogel exhibits excellent biocompatibility and undergoes a thermo‐sensitive phase transition, allowing for in situ gelation at body temperature and enabling localized, controlled drug release. The hydrogel was synthesized following a previously reported method.^[^ ^35^ ^]^

To prepare the final formulations, Se‐Met‐CD and Met‐CD were each dissolved in PBS to concentrations of 10.0 and 60.0 µg mL^−1^, respectively. These solutions were mixed with the PM polymer to a final polymer concentration of 6.0 wt.% and stirred at 0 °C for 72 h. The resulting injectable CD‐loaded hydrogels were designated as Se‐Met‐CD/Gel and Met‐CD/Gel. A blank hydrogel containing 6.0 wt.% PM was also prepared and referred to as Gel.

### Biodistribution Study in vivo

To investigate the in vivo biodistribution, Se‐Met‐CD was first conjugated with a Cy5.5‐NHS ester dye. Following dialysis and lyophilization, the resulting Se‐Met‐CD‐Cy5.5 conjugate was dissolved in PBS. This solution was then mixed with the PM polymer to a final polymer concentration of 6.0 wt.% and stirred at 0 °C for 72 h to form the final hydrogel formulation. For administration, the Co7/8 intervertebral disc of each rat was localized using DR. Subsequently, the hydrogel containing the Se‐Met‐CD‐Cy5.5 conjugate was administered via a single intradiscal injection (20.0 µL). Twenty‐four hours post‐injection, the animals were euthanized by transcardial perfusion. To perform transcardial perfusion, the thoracic cavity was opened by lifting the xiphoid process and making an incision through the diaphragm and sternum to fully expose the chest. A blunt‐tipped needle was inserted into the left ventricle via the cardiac apex, an incision was made in the right atrial appendage, and the circulatory system was perfused with PBS to remove residual blood until the effluent ran clear. Following perfusion, the heart, liver, spleen, lungs, kidneys, and the caudal vertebral column were harvested. The ex vivo fluorescence imaging of these organs and tissues was conducted using an in vivo imaging system.

### Animal Model and Surgical Procedure

The animal study was conducted in accordance with the regulations and guidelines of the Institutional Animal Care and Use Committee (IACUC) of the Changchun Institute of Applied Chemistry (IACUC Issue No. CIAC 2023[0116]). Thirty female Sprague‐Dawley rats (eight weeks old) were randomly assigned to five groups (*n* = 6 per group): Normal, Control, Gel, Met‐CD/Gel, and Se‐Met‐CD/Gel. The Normal group served as the healthy, unoperated control and therefore received no interventions.

For surgery, rats were anesthetized using inhaled isoflurane followed by an intraperitoneal injection of tribromoethanol. Once fully anesthetized, the rats were placed on a heating pad, and their tails were disinfected with povidone‐iodine. To precisely localize the surgical site, a two‐step procedure was employed: first, a landmark was identified ≈7.5 cm distal to the urethral opening to guide imaging. A DR scan was then performed, centered on this landmark, to accurately identify and confirm the Co7/8 intervertebral disc as the target level. A 21‐gauge (21G) sterile needle was inserted vertically into the Co7/8 intervertebral space to a depth of 5.5 mm. The needle was then rotated 720 ° and held in place for 30 s to induce disc injury. This procedure created an enhanced, more severe injury model, enabling more precise assessment of the therapeutic potential of the materials.

While the needle remained in position, a microsyringe was used to deliver an intradiscal injection. The Control group received 20.0 µL of PBS, while the Gel group was injected with 20.0 µL of the base hydrogel. The Se‐Met‐CD/Gel and Met‐CD/Gel groups received 20.0 µL of their respective formulations containing Se‐Met‐CD (10.0 µg mL^−1^) and Met‐CD (60.0 µg mL^−1^). The injection site was marked externally with black ink to aid in post‐surgical localization.

Therapeutic outcomes were evaluated at 4 and 8 weeks post‐operation. At each time point, three rats per group (*n* = 3) underwent DR and MRI evaluations. Following imaging, they were euthanized, and their caudal spine segments were harvested for further analysis.

### Radiographic and Magnetic Resonance Imaging Analysis

DR images were acquired to evaluate changes in the height of intervertebral discs. The DHI was calculated using a previously reported method.^[^ ^36^ ^]^ MRI was conducted using a T2‐weighted imaging sequence. The T2 signal intensity reflects the water content of NP, serving as a biomarker of disc hydration and degeneration. The severity of IVDD was graded using the Pfirrmann grading system, which classifies degeneration from Grade I (normal) to Grade V (severely degenerated). Grading is based on the signal intensity, internal structure homogeneity, border definition between the NP and the annulus fibrosus, and the disc height. Higher Pfirrmann grades indicate more advanced degeneration.

### Histological Staining and Analysis

After euthanasia, the tails of rats were excised, and the spinal columns were carefully isolated from surrounding soft tissues using surgical tools. Motion segments, including the intervertebral discs and adjacent vertebral bodies, were harvested and subjected to decalcification and dehydration for 15 days. The processed specimens were then embedded in paraffin and sectioned for further analysis.

The processed specimens were stained with H&E and SOFG to evaluate tissue morphology and extracellular matrix composition. The degree of disc degeneration was quantified using a previously reported histological grading scale.^[^ ^37^ ^]^ This scoring system evaluates the morphology of the anulus fibrosus, the clarity of the boundary between the anulus fibrosus and NP, NP cellularity, and the condition of the NP matrix. Each parameter is individually scored, with a total score ranging from 0 to 12. Higher scores reflect more severe IVDD.

### Statistical Analysis

All data are presented as the mean ± standard deviation (SD) of at least three independent experiments. Statistical analysis was performed using IBM SPSS Statistics (version 27.0.1, IBM Corp., Armonk, NY, USA). For comparisons between two groups, Student's *t*‐test was used. For comparisons among three or more groups, a one‐way analysis of variance (ANOVA) was followed by Tukey's post hoc test. A *p*‐value of less than 0.05 was considered statistically significant. Significance levels were denoted as follows: ^*^ indicates *p* < 0.05, ^**^ indicates *p* < 0.01, ^***^ indicates *p* < 0.001, and ^****^ indicates *p* < 0.0001. Differences with *p* > 0.05 were considered not significant (NS).

## Conflict of Interest

The authors declare no conflict of interest.

## Author Contributions

Q.Z. performed conceptualization, validation, formal analysis, investigation, resources, wrote the original draft, and reviewed and edited the final manuscript. Z.L. performed the investigation, resources, and reviewed and edited the final manuscript. Y.S. reviewed and edited the final manuscript. C.F. performed conceptualization, supervision, project administration, acquired funding acquisition, and reviewed and edited the final manuscript. J.D. performed conceptualization, supervision, project administration, acquired funding acquisition, and reviewed and edited the final manuscript.

## Supporting information



Supporting Information

## Data Availability

The data that support the findings of this study are available from the corresponding author upon reasonable request.

## References

[1] N. Fine , S. Lively , C. A. Séguin , A. V. Perruccio , M. Kapoor , R. Rampersaud , Nat. Rev. Rheumatol. 2023, 19, 136. 36702892 10.1038/s41584-022-00888-z

[2] V. Francisco , J. Pino , M. González‐Gay , F. Lago , J. Karppinen , O. Tervonen , A. Mobasheri , O. Gualillo , Nat. Rev. Rheumatol. 2022, 18, 47. 34845360 10.1038/s41584-021-00713-z

[3] U. Zehra , M. Tryfonidou , J. C. Iatridis , S. Illien‐Jünger , F. Mwale , D. Samartzis , Nat. Rev. Rheumatol. 2022, 18, 352. 35534553 10.1038/s41584-022-00783-7 PMC9210932

[4] W. Jiang , J. D. Glaeser , G. Kaneda , J. Sheyn , J. T. Wechsler , S. Stephan , K. Salehi , J. L. Chan , W. Tawackoli , P. Avalos , C. Johnson , C. Castaneda , L. E. A. Kanim , T. Tanasansomboon , J. E. Burda , O. Shelest , H. Yameen , T. G. Perry , M. Kropf , J. M. Cuellar , D. Seliktar , H. W. Bae , L. S. Stone , D. Sheyn , Sci. Transl. Med. 2023, 15, adg7020. 10.1126/scitranslmed.adg7020 PMC12083434 38055799

[5] J. Wang , Y. Jiang , C. Zhu , Z. Liu , L. Qi , H. Ding , J. Wang , Y. Huang , Y. Li , Y. Song , G. Feng , L. Zhang , L. Liu , Bioact. Mater. 2024, 40, 1. 38873262 10.1016/j.bioactmat.2024.05.044 PMC11167444

[6] H. H. Genedy , P. Humbert , B. Laoulaou , B. Le Moal , M. Fusellier , C. Passirani , C. L. Visage , J. Guicheux , É. Lepeltier , J. Clouet , Adv. Drug Deliv. Rev. 2024, 207, 115214. 38395361 10.1016/j.addr.2024.115214

[7] F. Li , M. Chen , M. Zhang , S. Chen , M. Qu , S. He , L. Wang , X. Wu , G. Xiao , J. Orthop. Translat. 2025, 51, 145. 40129609 10.1016/j.jot.2025.01.006 PMC11930658

[8] H. Swahn , J. Mertens , M. Olmer , K. Myers , T. S. Mondala , P. Natarajan , S. R. Head , O. Alvarez‐Garcia , M. K. Lotz , Adv. Sci. 2024, 11, 2309032. 10.1002/advs.202309032 PMC11077672 38403470

[9] M. G. Fehlings , J. H. Badhiwala , H. Ahn , H. F. Farhadi , C. I. Shaffrey , A. Nassr , P. Mummaneni , P. M. Arnold , W. B. Jacobs , K. D. Riew , M. Kelly , D. S. Brodke , A. R. Vaccaro , A. S. Hilibrand , J. Wilson , J. S. Harrop , S. T. Yoon , K. D. Kim , D. R. Fourney , C. Santaguida , E. M. Massicotte , B. Kopjar , Lancet Neurol. 2021, 20, 98. 33357512 10.1016/S1474-4422(20)30407-5

[10] H. J. Wilke , V. Sciortino , Biomaterials 2025, 312, 122717. 39121730 10.1016/j.biomaterials.2024.122717

[11] Z. Liu , Q. Zhang , Y. Li , G. Wang , C. Fu , Y. Sun , J. Ding , Biomaterials 2025, 324, 123426. 40472408 10.1016/j.biomaterials.2025.123426

[12] K. Sun , Y. Shi , C. Yan , S. Wang , L. Han , F. Li , X. Xu , Y. Wang , J. Sun , Z. Kang , J. Shi , Adv. Sci. 2025, 12, 2416149. 10.1002/advs.202416149 PMC12140309 40171826

[13] X. Wang , Z. Guo , L. Chen , J. Sun , K. Y. H. Kwan , M. Jones , Y. M. Li , Y. Hu , X. Wang , P. Makvandi , X. Wang , Q. Qian , Y. Zhou , A. Wu , Adv. Sci. 2025, 12, 2500128. 10.1002/advs.202500128 PMC12199421 40145798

[14] S. Liu , Y. Hu , W. Xu , W. Liu , B. Wang , X. Zeng , Z. Shao , C. Yang , L. Xiong , X. Cai , Autophagy 2025, 21, 979. 39675125 10.1080/15548627.2024.2440844 PMC12013417

[15] X. Chen , A. Zhang , K. Zhao , H. Gao , P. Shi , Y. Chen , Z. Cheng , W. Zhou , Y. Zhang , Ageing Res. Rev. 2024, 98, 102323. 38734147 10.1016/j.arr.2024.102323

[16] Y. Huang , J. Sun , S. Li , Y. Shi , L. Yu , A. Wu , X. Wang , Radic. Biol. Med. 2024, 225, 98. 10.1016/j.freeradbiomed.2024.10.001 39366471

[17] K. Sun , C. Yan , X. Dai , Y. Shi , F. Li , L. Chen , J. Sun , Y. Chen , J. Shi , Adv. Mater. 2024, 36, 2313248. 10.1002/adma.202313248 38299823

[18] L. Jiang , H. Cai , W. Zhou , Z. Li , L. Zhang , H. Bi , Adv. Mater. 2023, 35, 2210776. 10.1002/adma.202210776 36645339

[19] Y. Yang , B. Wang , X. Zhang , H. Li , S. Yue , Y. Zhang , Y. Yang , M. Liu , C. Ye , P. Huang , X. Zhou , Adv. Mater. 2023, 35, 2211337. 10.1002/adma.202211337 37025038

[20] D. Wang , Y. Hou , L. Ren , Y. Jiang , Y. Meng , R. Ma , S. Wang , Z. Liu , X. Li , F. Cui , T. Li , J. Li , Food. Chem. 2025, 491, 145191. 40580597 10.1016/j.foodchem.2025.145191

[21] M. J. Nasim , M. M. Zuraik , A. Y. Abdin , Y. Ney , C. Jacob , Antioxidants 2021, 10, 882. 34072794 10.3390/antiox10060882 PMC8229699

[22] B. Khatri , S. Raghunathan , S. Chakraborti , R. Rahisuddin , S. Kumaran , R. Tadala , P. Wagh , U. D. Priyakumar , J. Chatterjee , Angew. Chem. Int. Ed. 2021, 60, 24870. 10.1002/anie.202110978 34519402

[23] F. Li , T. Li , C. Sun , J. Xia , Y. Jiao , H. Xu , Angew. Chem. Int. Ed. 2017, 56, 9910. 10.1002/anie.201705989 28643462

[24] Z. Yang , T. Xu , S. Zhang , H. Li , Y. Ji , X. Jia , J. Li , Nano. Res. 2023, 16, 5401. 36405981 10.1007/s12274-022-5107-7 PMC9643953

[25] M. Alafeef , I. Srivastava , T. Aditya , D. Pan , Small 2024, 20, 2303937. 10.1002/smll.202303937 37715112

[26] G. Barchielli , A. Capperucci , D. Tanini , Antioxidants 2022, 11, 251. 35204134 10.3390/antiox11020251 PMC8868242

[27] W. Hou , H. Xu , J. Med. Chem. 2022, 65, 4436. 35244394 10.1021/acs.jmedchem.1c01859

[28] H. Li , J. Guo , A. Liu , Y. Chen , Y. He , J. Qu , W. Yan , J. Song , Adv. Funct. Mater. 2023, 33, 2212141.

[29] A. Truskewycz , H. Yin , N. Halberg , D. T. H. Lai , A. S. Ball , V. K. Truong , A. M. Rybicka , I. Cole , Small 2022, 18, 2106342. 10.1002/smll.202106342 35088534

[30] P. Bu , R. Peng , J. Zhang , Z. He , S. Gou , X. Liu , X. Qiu , B. Zhou , W. Meng , H. Fu , H. Zhu , B. Gao , M. Serda , F. Li , Q. Feng , K. Cai , Adv. Mater. 2025, 37, 2411290. 10.1002/adma.202411290 39713901

[31] A. T. Dinkova‐Kostova , I. M. Copple , Trends Pharmacol. Sci. 2023, 44, 137. 36628798 10.1016/j.tips.2022.12.003

[32] J. Zhou , J. Qiu , Y. Song , T. Liang , S. Liu , C. Ren , X. Song , L. Cui , Y. Sun , Cell Death. Dis. 2023, 14, 94. 36755014 10.1038/s41419-023-05634-1 PMC9908978

[33] A. Kamali , R. Ziadlou , G. Lang , J. Pfannkuche , S. Cui , Z. Li , R. G. Richards , M. Alini , S. Grad , Theranostics 2021, 11, 27. 33391459 10.7150/thno.48987 PMC7681102

[34] Y. Dou , Y. Zhang , Y. Liu , X. Sun , X. Liu , B. Li , Q. Yang , Bone Res. 2025, 13, 15. 39848963 10.1038/s41413-024-00397-7 PMC11758090

[35] Q. Luo , J. Sun , Z. Li , B. Liu , J. Ding , Chin. Chem. Lett. 2025, 36, 110433.

[36] S. Liang , N. Li , J. Zhan , Z. Li , C. Tie , Y. Zhu , H. Guo , L. Ke , J. Li , Z. Xu , P. Zhang , W. Cheng , J. Orthop. Surg. Res. 2024, 19, 632. 39375759 10.1186/s13018-024-05110-2 PMC11457380

[37] W. Bu , Y. Shi , X. Huang , S. Wu , L. Jiang , C. Pan , D. Li , Z. Xu , H. Wang , H. Chen , J. Du , J. Nanobiotechnol. 2024, 22, 412. 10.1186/s12951-024-02683-2 PMC11241859 38997713

