# Supplementary material for: Antioxidant Carbon Dot of Selenomethionine Alleviates Oxidative Stress in Intervertebral Disc Degeneration
Source: Adv Sci (Weinh). 2025 Sep 17;12(45):e14217. doi: 10.1002/advs.202514217 (PMC12677702; doi:10.1002/advs.202514217)
Supplement: Supplementary file 1 — Supporting Information [file ADVS-12-e14217-s001.docx]

Supporting Information

**Antioxidant Carbon Dot of Selenomethionine Alleviates Oxidative Stress in Intervertebral Disc Degeneration**

*Qingzheng Zhang, Zongtai Liu, Yirong Sun, Changfeng Fu,* and Jianxun Ding**

Dr. Q. Zhang, Dr. Z. Liu, Prof. C. Fu

Department of Spine Surgery, Center of Orthopedics

The First Hospital of Jilin University

1 Xinmin Street, Changchun 130061, P. R. China

E-mail: fucf@jlu.edu.cn

Prof. Y. Sun, Prof. J. Ding

State Key Laboratory of Polymer Science and Technology

Changchun Institute of Applied Chemistry, Chinese Academy of Sciences

5625 Renmin Street, Changchun 130022, P. R. China

E-mail: jxding@ciac.ac.cn

Prof. J. Ding

School of Applied Chemistry and Engineering

University of Science and Technology of China

96 Jinzhai Road, Hefei 230026, P. R. China

**Experiment Section**

*Materials:* L-Selenomethionine was purchased from Shanghai Anaji Chemical Reagent Co., Ltd. (Shanghai, P. R. China). L-Methionine and L-valine were obtained from Aladdin Reagent Co., Ltd. (Shanghai, P. R. China). Methoxy poly(ethylene glycol) (mPEG; number-average molecular weight (*M*_n_) = 2000 g/mol) was purchased from Sigma-Aldrich (Shanghai, P. R. China). Deuterated chloroform (CDCl_3_), deuterated trifluoroacetic acid (TFA-*d*), and deuterium oxide (D_2_O) were purchased from Cambridge Isotope Laboratories, Inc. (Andover, MA, USA). Tetrahydrofuran (THF), *N*,*N*-dimethylformamide (DMF), and dichloromethane (DCM) were obtained from Energy Chemical (Anhui, P. R. China). Triphosgene was obtained from Duodian Chemical Co., Ltd. (Shanghai, P. R. China). All other chemical reagents and solvents were purchased from Sinopharm Chemical Reagent Co., Ltd. (Shanghai, P. R. China) and used without further purification.

Quinine sulfate was purchased from the National Institute of Metrology (Beijing, P. R. China). The Total Antioxidant Capacity Assay Kit (ABTS method) was purchased from Beyotime Biotechnology Co., Ltd. (Shanghai, P. R. China). The 2,2-diphenyl-1-picrylhydrazyl (DPPH) radical scavenging assay kit was obtained from Solarbio Life Sciences (Beijing, P. R. China). Calf serum was purchased from Siqing Bioengineering Materials Co., Ltd. (Tianjin, P. R. China). DMEM/F12 Cell Culture Medium, Calcein-AM/propidium iodide (PI) Cell Viability Assay Kit, penicillin/streptomycin solution, hematoxylin and eosin (H&E) Staining Kit, and Safranin O**/**Fast Green Staining Kit were purchased from Servicebio Technology Co., Ltd. (Wuhan, P. R. China). The Cell Counting Kit-8 (CCK-8) Cell Proliferation Assay Kit and Collagenase Type II were obtained from Biosharp Biotechnology Co., Ltd. (Hefei, P. R. China). The reactive oxygen species (ROS) detection probe and Annexin V-fluorescein isothiocyanate (FITC)/PI Apoptosis Detection Kit were provided by Meilun Biotechnology Co., Ltd. (Dalian, P. R. China).

*Characterizations:* Transmission electron microscopy (TEM) was performed using a JEOL JEM-1011 microscope from JEOL Co., Ltd. (Tokyo, Japan). Fourier-transform infrared (FT-IR) spectra were recorded using a Bio-Rad FTS-600 instrument from Bio-Rad Laboratories Inc. (Cambridge, MA, USA). Proton Nuclear Magnetic Resonance spectra were recorded using a Bruker spectrometer from Bruker Biospin Co., Ltd. (Rheinstetten, Germany). Confocal laser scanning microscopy (CLSM) was performed using a ZEISS LSM 780 system from Carl Zeiss AG (Jena, Germany). Absorbance for the CCK-8 assay was measured using a Bio-Rad microplate reader from Bio-Rad Laboratories Inc. (Hercules, CA, USA). X-ray imaging was performed using a PerfoX 3000B-1 digital X-ray radiography system from Shenzhen Browiner Tech Co., Ltd. (Shenzhen, P. R. China). Magnetic resonance imaging (MRI) was performed on a uMR 580 MRI scanner from United Imaging Healthcare Co., Ltd. (Shanghai, P. R. China).

*Isolation and Culture of Rat Nucleus Pulposus Cells:* Fifteen female Sprague-Dawley rats (10 weeks old) were euthanized via isoflurane overdose and disinfected in 75% (*V*/*V*) ethanol. Under aseptic conditions in a laminar flow hood, nucleus pulposus (NP) tissues were dissected and transferred into DMEM/F12 medium. Tissues were digested with 0.5% (*W/V*) collagenase type II at 37 °C for 3 h, followed by filtration through a 70 μm cell strainer. The cell suspension was centrifuged at 1200 rpm for 5 min, and the pellet was resuspended in complete DMEM/F12 medium containing 10% (*V*/*V*) fetal bovine serum (FBS) and 2% (*V*/*V*) penicillin/streptomycin. Cells were cultured in a standard cell incubator, with the first medium change occurring on day 4, and subsequent passages performed every two days. Nucleus pulposus cells (NPCs) at passages 3−10 were used in all experiments. Collagen type II (Col-II) immunostaining was used to verify NPC identity after initial isolation.

*Cell Viability Analysis:* Cytotoxicity was evaluated using the CCK-8 assay. NPCs were seeded in 96-well plates at a density of 7,000 cells per well and incubated for 24 h at 37 °C in a 5% (*V*/*V*) carbon dioxide (CO_2_) atmosphere. The cells were then treated for 24 h with DMEM/F12 medium containing varying concentrations (0, 2.5, 5.0, 10.0, 20.0, 40.0, 60.0, 80.0, and 100.0 μg mL^−1^) of the respective carbon dots (CDs) or their precursor amino acids. Following treatment, 10% (*V/V*) CCK-8 reagent diluted in DMEM/F12 medium was added, and the absorbance at 450 nm was measured using a microplate reader. To assess the ability of the CDs and their precursor amino acids to counteract oxidative stress, NPCs were similarly seeded in 96-well plates (7,000 cells/well) and incubated for 24 h. The cells were then treated for 24 h with medium containing various concentrations of CDs or precursor amino acids, along with hydrogen peroxide (H_2_O_2_) at a final concentration of 200.0 μM. Afterward, cell viability was determined using the CCK-8 assay as described above.

*Cell Uptake of Carbon Dots by Nucleus Pulposus Cells:* NPCs were seeded at a density of 60,000 cells per dish in glass-bottom confocal dishes and incubated for 24 h at 37 °C in a 5% (*V*/*V*) CO_2_ atmosphere. To ensure a robust fluorescence signal for imaging, the cells were then incubated with DMEM/F12 medium containing Se-Met-CD, Met-CD, or Val-CD at a concentration of 100.0 μg mL^−1^. At time points of 3, 6, 9, and 12 h, dishes were individually imaged using a CLSM to observe intracellular fluorescence. An excitation wavelength of 488 nm was used.

*Calcein-AM/PI Live/Dead Cell Staining:* Live/dead staining was conducted using a Calcein-AM/PI Cell Viability/Cytotoxicity Assay Kit. NPCs were seeded into 96-well plates at a density of 8,000 cells per well and incubated for 24 h. The cells were then treated with DMEM/F12 medium containing Se-Met-CD at 10.0 μg mL^−1^, Met-CD at 60.0 μg mL^−1^, or Val-CD at 100.0 μg mL^−1^, along with H_2_O_2_ at a final concentration of 300.0 μM. After 8 h of incubation, the medium was removed, and the cells were gently washed with phosphate-buffered saline (PBS). Then, 100.0 μL of Calcein-AM/PI working solution was added to each well, followed by incubation at 37 °C for 15−30 min in the dark. Fluorescence images were captured using a fluorescence microscope. Live cells emitted green fluorescence from Calcein-AM, whereas dead cells showed red fluorescence from PI staining.

*Apoptosis Analysis by Annexin V-FITC/PI Double Staining:* Early apoptosis under oxidative stress was assessed by flow cytometry using a FITC/PI Apoptosis Detection Kit. NPCs were seeded in 12-well plates at a density of 2.0 × 10^5^ cells per well and incubated for 7 h to allow for cell attachment. Cells were then treated with medium containing Se-Met-CD at a concentration of 10.0 μg mL^−1^, Met-CD at 60.0 μg mL^−1^, Val-CD at 100.0 μg mL^−1^, or their corresponding precursor amino acids at the same respective concentrations, and co-exposed to H_2_O_2_ at a final concentration of 200.0 μM. After 12 h of incubation, cells were rinsed with PBS and detached using EDTA-free trypsin for 5 min. The cell suspension was transferred to flow cytometry tubes, centrifuged at 1100 rpm for 5 min, and washed once with PBS. Cells were resuspended and stained with FITC and PI according to the kit instructions for 15 min at room temperature. Samples were analyzed using a flow cytometer, and data were processed with FlowJo software (version 10.9.0, BD Life Sciences, Ashland, OR, USA).

*Quantitative Real-Time PCR Analysis:* NPCs were seeded into 12-well plates at a density of 1.0 × 10^5^ cells per well and incubated for 24 h. Cells were then assigned to five groups: Normal (no treatment), Control (treated with 200.0 μM H_2_O_2_), Se-Met-CD (10.0 μg mL^−1^ Se-Met-CD and 200.0 μM H_2_O_2_), Met-CD (60.0 μg mL^−1^ Met-CD and 200.0 μM H_2_O_2_), and Val-CD (100.0 μg mL^−1^ Val-CD and 200.0 μM H_2_O_2_). After 24 h of treatment, total RNA was extracted and submitted to Servicebio (Wuhan, China) for Quantitative Real-Time PCR analysis. Glyceraldehyde 3-phosphate dehydrogenase was used as the internal control.

*Western Blot Analysis:* Following the same treatment protocol described above, cells were lysed using RIPA buffer supplemented with protease and phosphatase inhibitors. The lysates were centrifuged at 12,000 rpm for 15 min at 4 °C, and the supernatants were collected. Protein concentrations were determined using the BCA protein assay. Equal amounts of protein were resolved by sodium dodecyl sulfate–polyacrylamide gel electrophoresis and transferred onto polyvinylidene difluoride membranes. Membranes were blocked with 5% (*W/V*) non-fat milk for 1 h at room temperature, followed by incubation with primary antibodies overnight at 4 °C. After washing, membranes were incubated with horseradish peroxidase-conjugated secondary antibodies for 1 h at room temperature. Signals were visualized using an enhanced chemiluminescence system, and band intensities were quantified using ImageJ software (version 1.8.0, National Institutes of Health, Bethesda, MD, USA; https://imagej.net/).

*Synthesis of Hydrogel:* Briefly, mPEG was first reacted with *p*-toluenesulfonyl chloride to convert its terminal hydroxyl group into a tosylate. The intermediate was then subjected to nucleophilic substitution with aqueous ammonia to generate amino-terminated mPEG (mPEG-NH_2_), which served as the macroinitiator for the subsequent polymerization. In parallel, L-methionine *N*-carboxyanhydride (Met NCA) monomer was prepared by reacting Met with triphosgene in an anhydrous solvent. Ring-opening polymerization was initiated by adding mPEG-NH_2_ to the Met NCA under strictly anhydrous and anaerobic conditions. The amino terminus of mPEG attacks the NCA ring, initiating chain growth and forming the poly(L-methionine) block. Following completion of the reaction, the resulting copolymer was purified by precipitation in diethyl ether, dialyzed against deionized water, and lyophilized to yield a white solid.


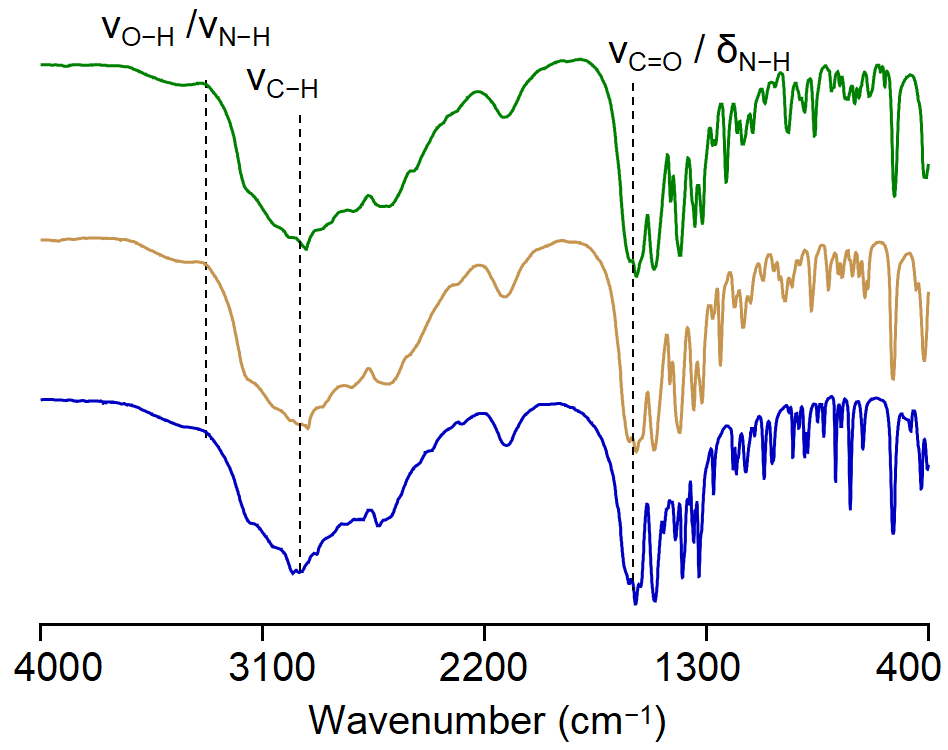


**Figure S1.** FT-IR spectra of amino acids. Val is represented in blue, Met is represented in brown, and Se-Met is represented in green.


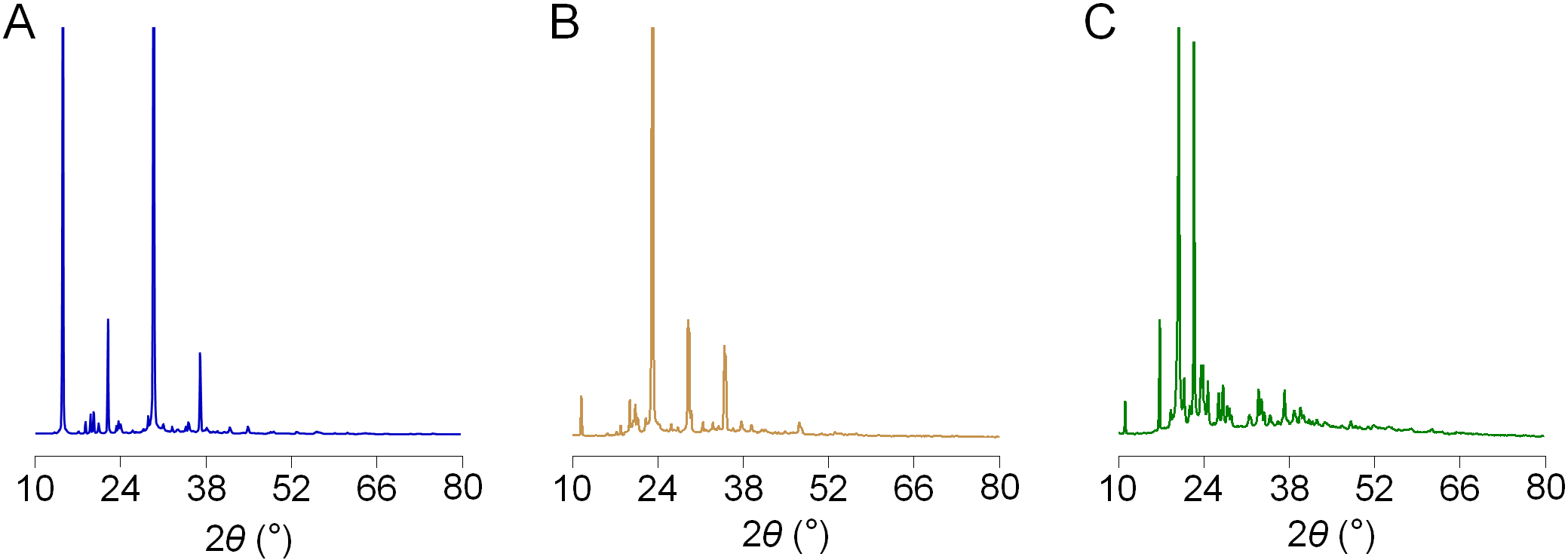


**Figure S2.** XRD patterns of amino acids. Val is represented in blue (A), Met in brown (B), and Se-Met in green (C).


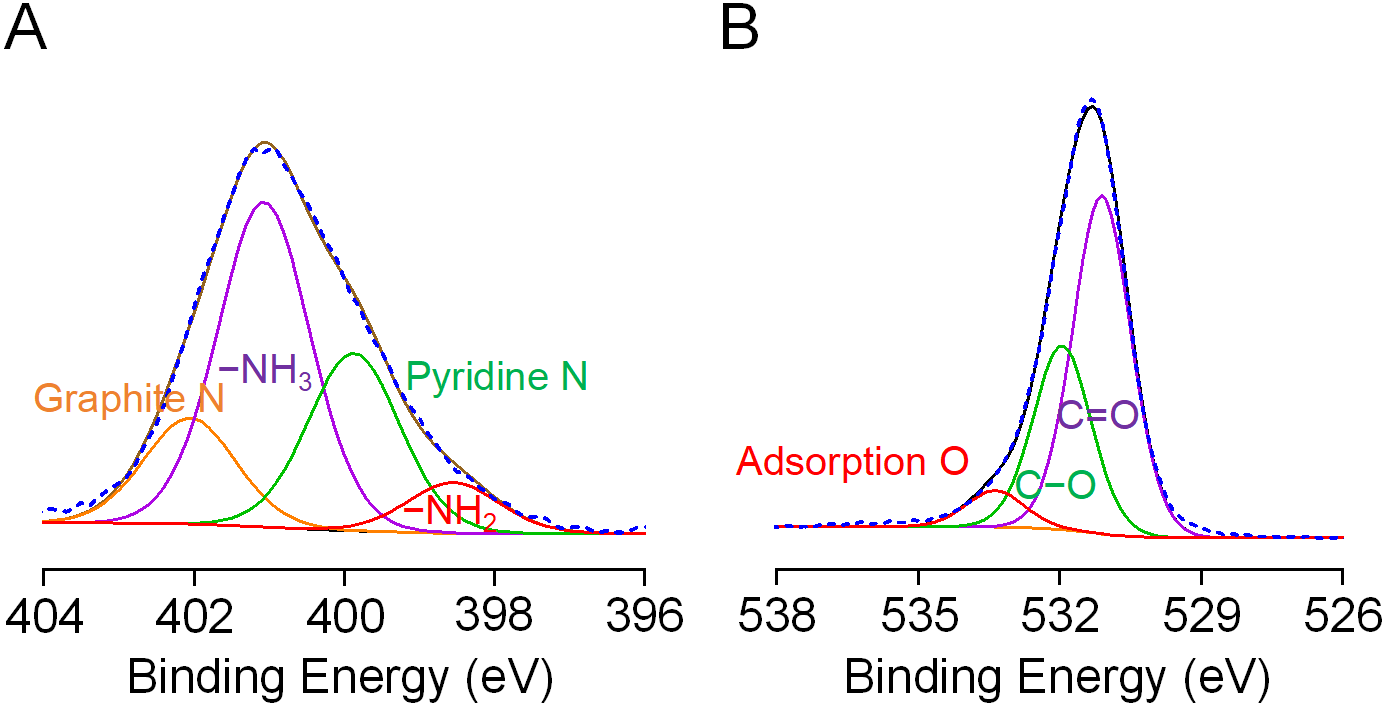


**Figure S3.** High-resolution XPS spectra of Val-CD. N 1s (A) and O 1s spectra (B). The solid lines represent the experimental data.


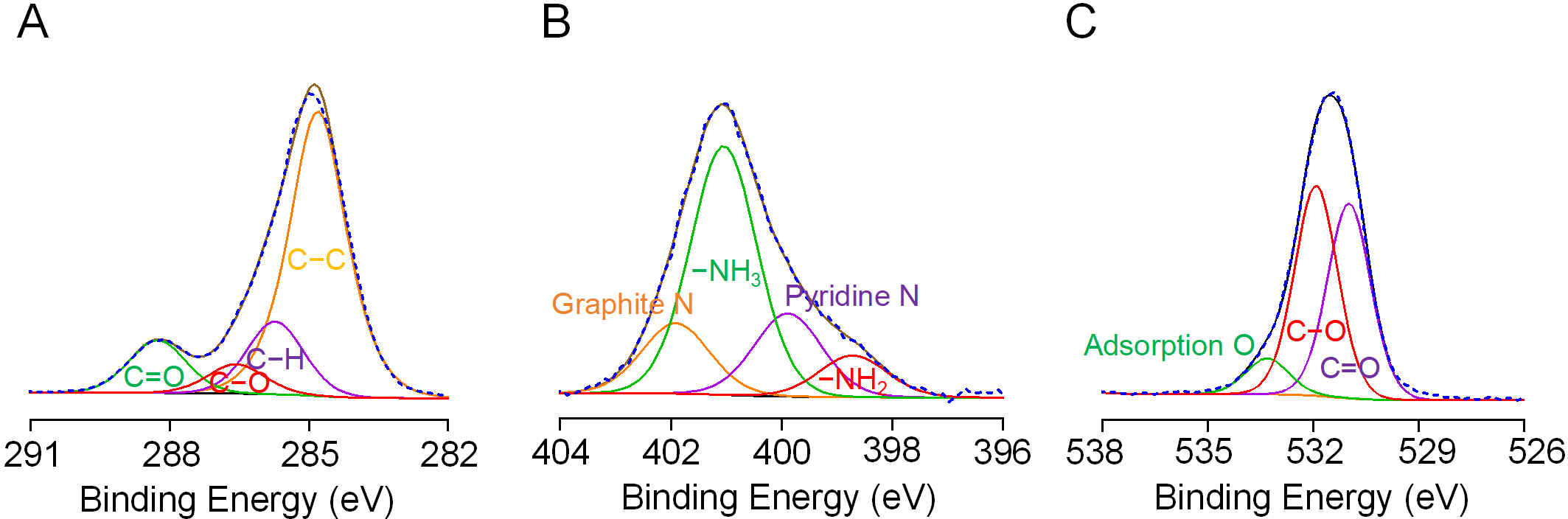


**Figure S4.** High-resolution XPS spectra of Met-CD. C 1s (A), N 1s (B), and O 1s spectra (C).


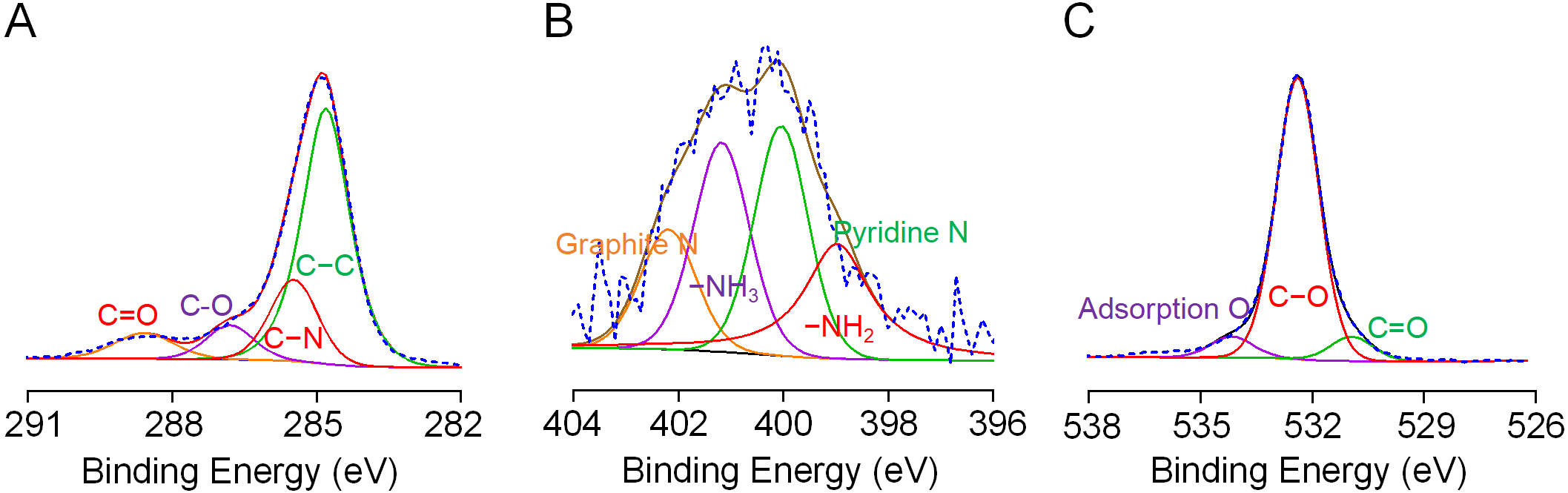


**Figure S5.** High-resolution XPS spectra of Se-Met-CD. C 1s (A), N 1s (B), and O 1s spectra (C).


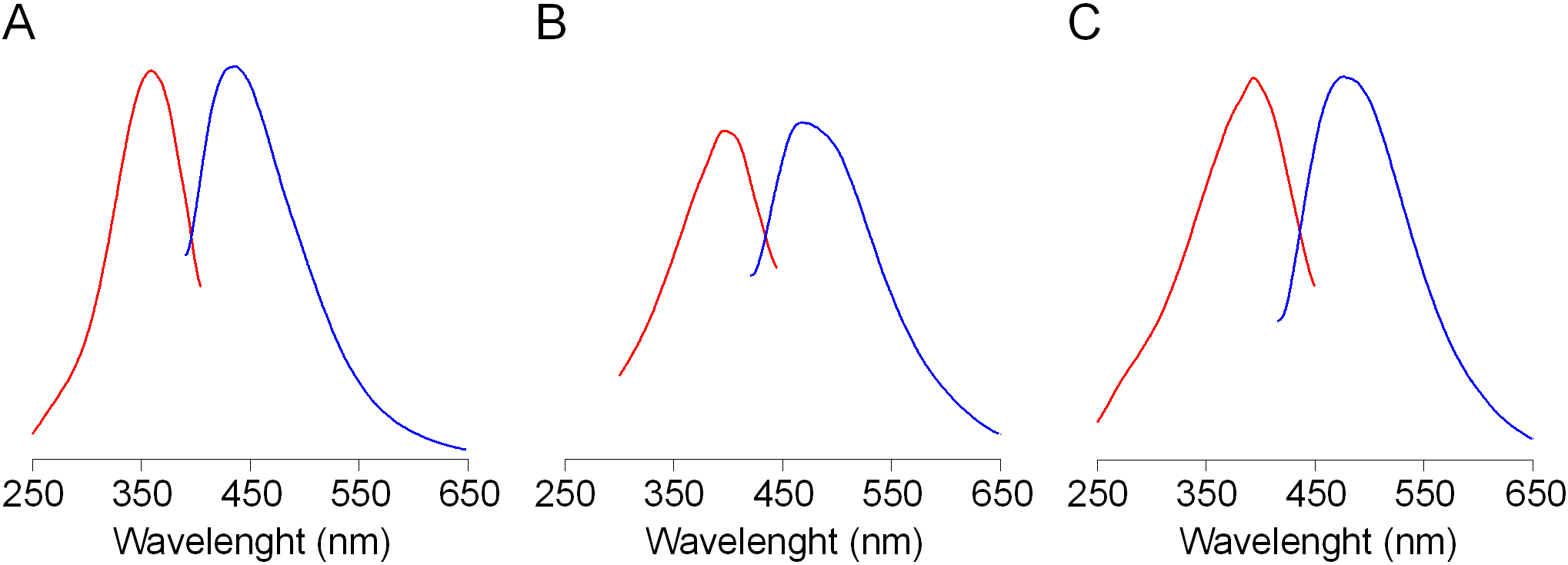


**Figure S6.** Excitation and emission peaks of Val-CD (A), Met-CD (B), and Se-Met-CD (C). The excitation peak is represented in red, and the emission peak is represented in blue.


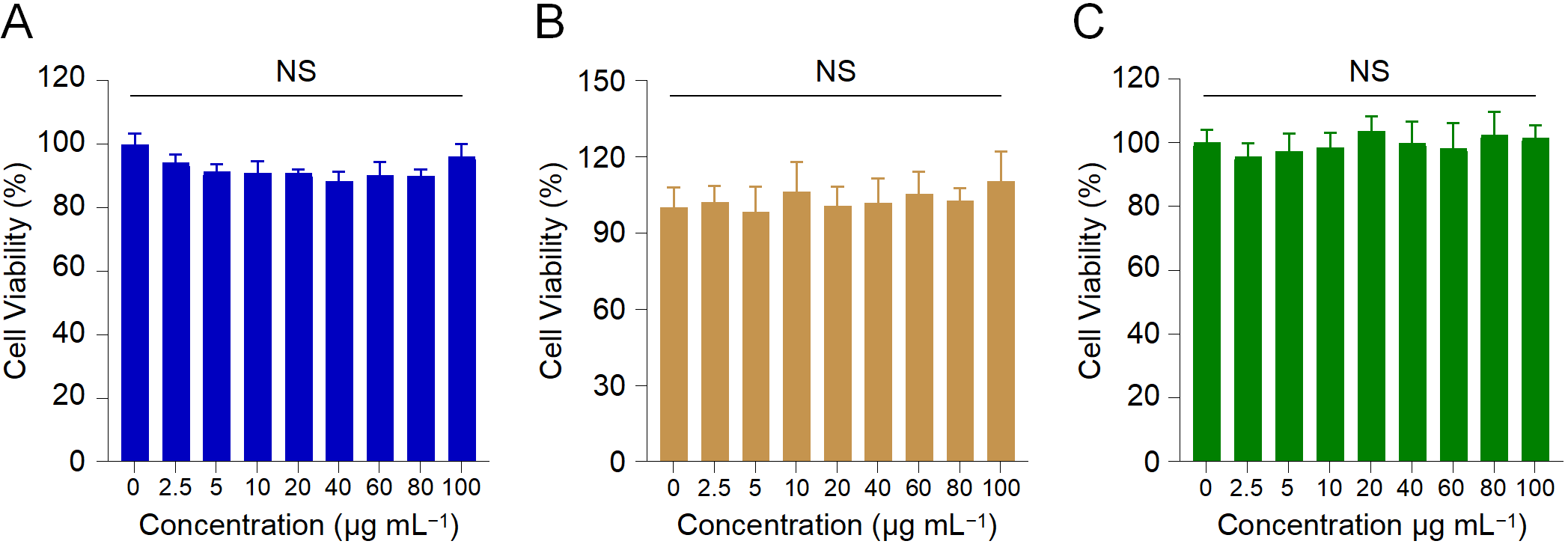


**Figure S7.** CCK-8 assay results of cell viability after treatment with CDs at various concentrations (0−100.0 μg mL^−1^). Val-CD is represented in blue (A), Met-CD in brown (B), and Se-Met-CD in green (C). Data are represented as mean ± standard deviation (SD; *n* = 6; NS, no significant difference; **p* < 0.05, ***p* < 0.01, ****p* < 0.001, *****p* < 0.0001).


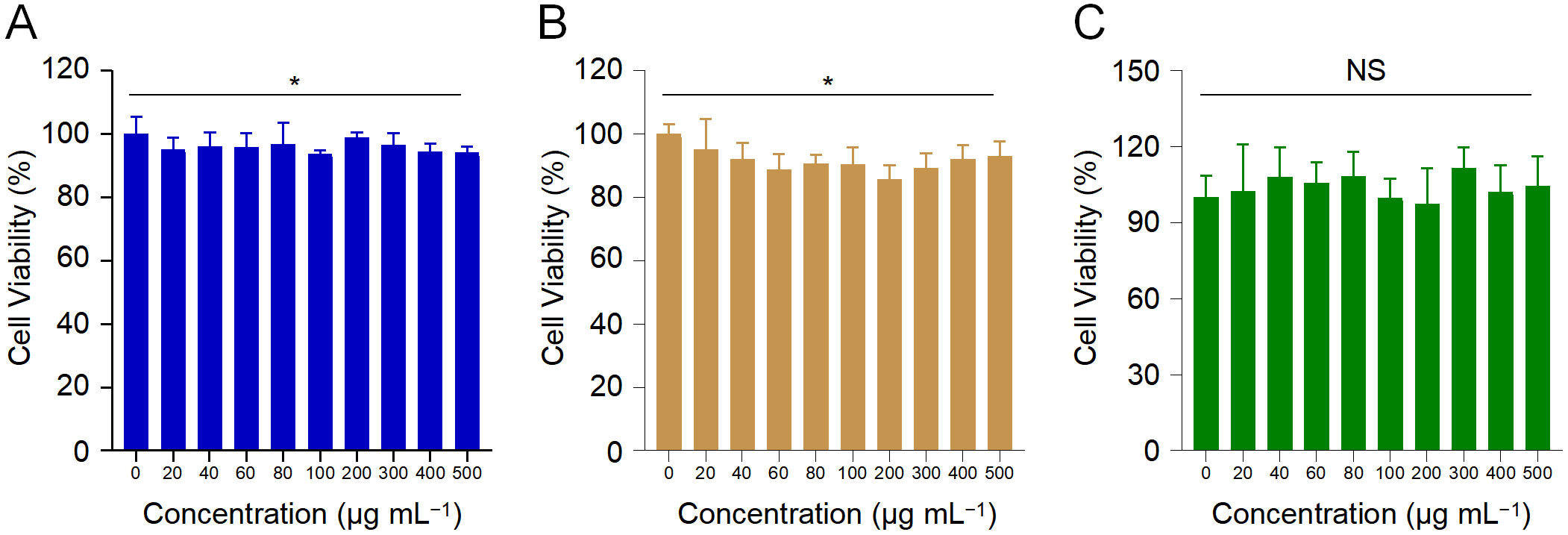


**Figure S8.** CCK-8 assay results of cell viability after treatment with amino acids at various concentrations (0−100.0 μg mL^−1^). Val is represented in blue (A), Met in brown (B), and Se-Met in green (C). Data are represented as mean ± SD (*n* = 6; NS, no significant difference; **p* < 0.05, ***p* < 0.01, ****p* < 0.001, *****p* < 0.0001).


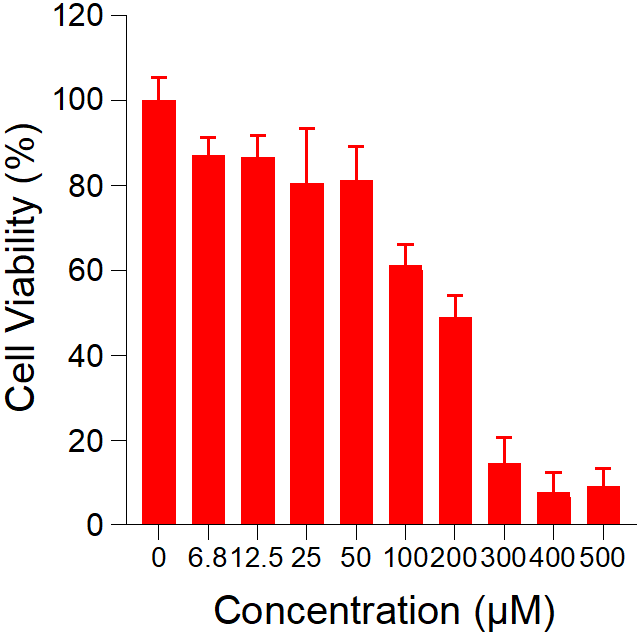


**Figure S9.** CCK-8 assay results of dose-dependent cytotoxicity of H_2_O_2_ on NPCs. Data are represented as mean ± SD (*n* = 6).


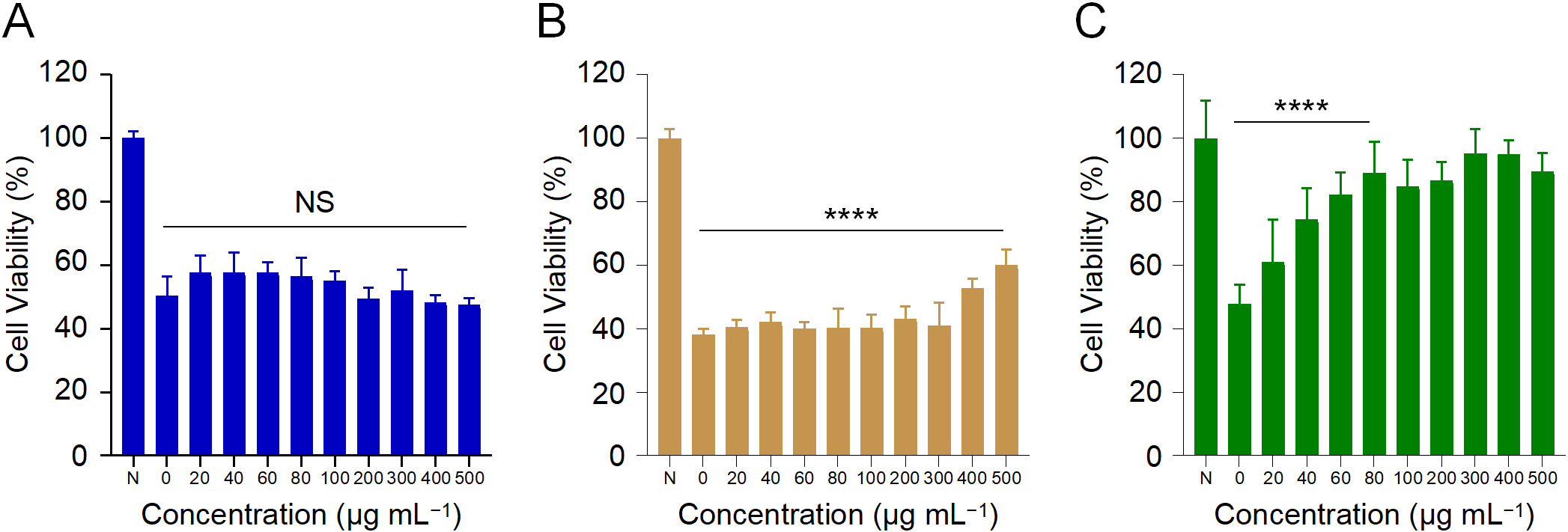


**Figure S10.** CCK-8 assay results of cell viability of NPCs after 24 h of co-incubation with various concentrations of amino acids in the presence of 200.0 μM H_2_O_2_. Val (A) is represented in blue, Met in brown (B), and Se-Met in green (C). Data are represented as mean ± SD (*n* = 6; NS, no significant difference; **p* < 0.05, ***p* < 0.01, ****p* < 0.001, *****p* < 0.0001).


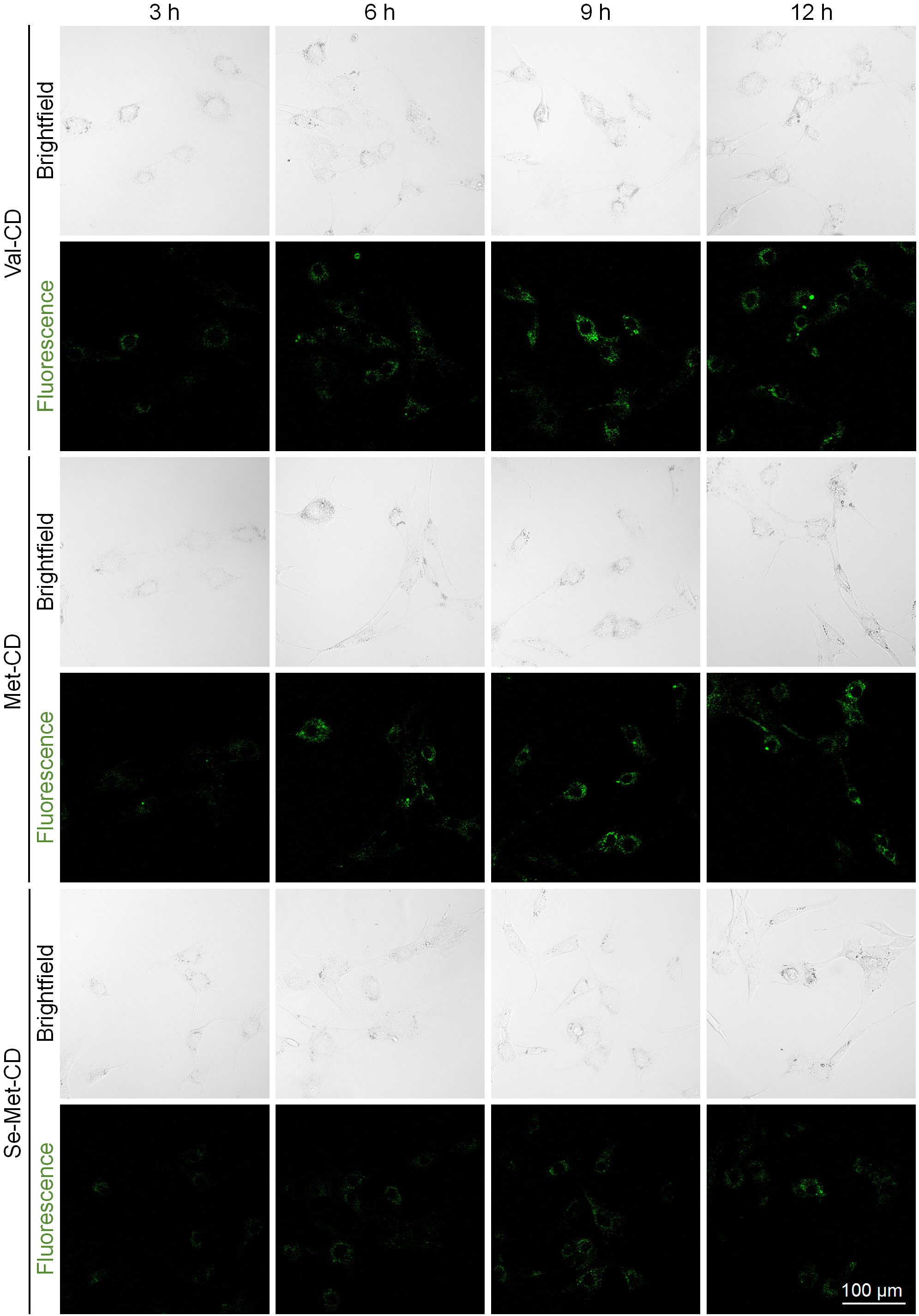


**Figure S11.** Brightfield and fluorescence microscopy images demonstrating cell uptake of Val-CD, Met-CD, and Se-Met-CD.


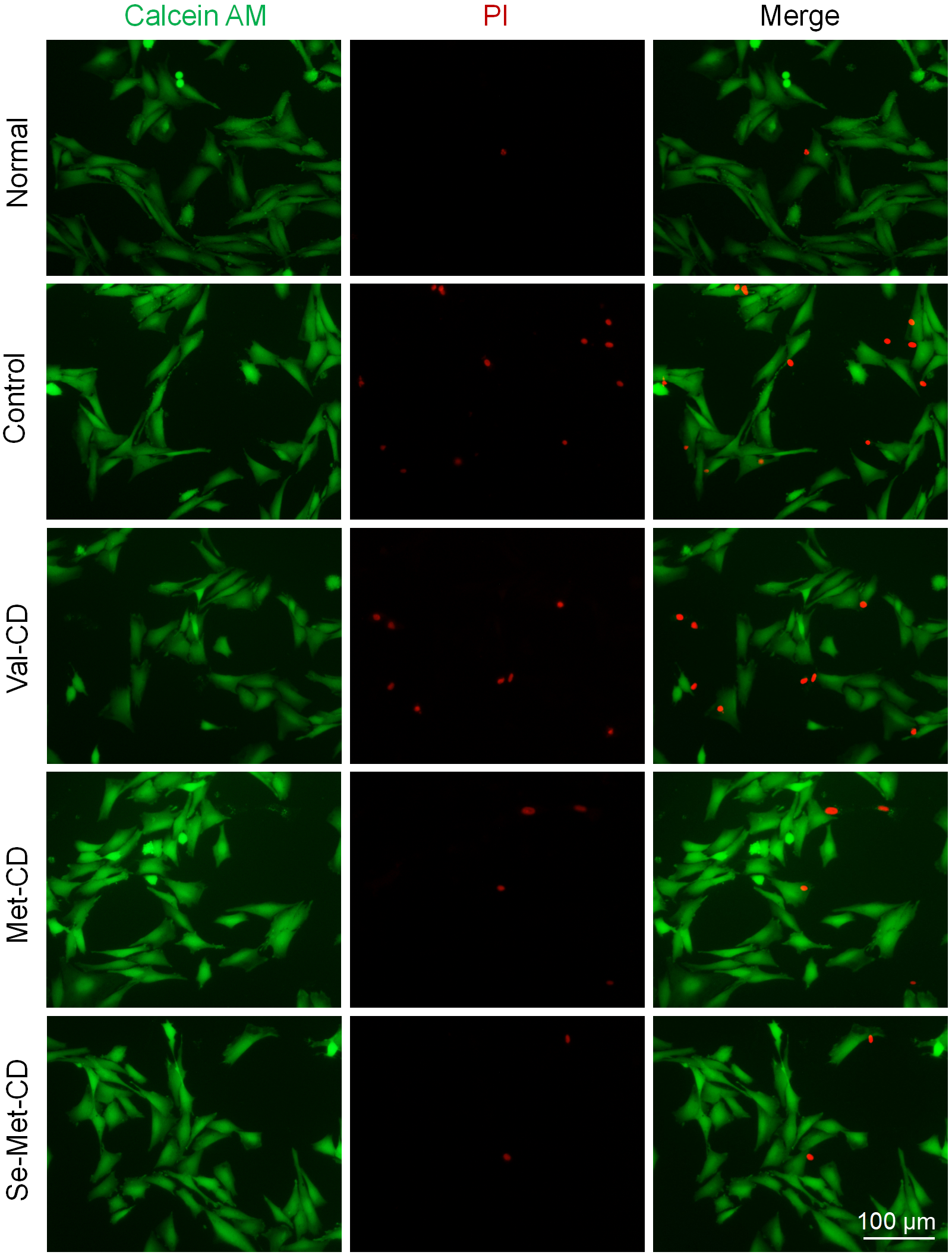


**Figure S12.** Calcein AM/PI staining images of cells pretreated with CDs in presence of H_2_O_2_. Live cells show green fluorescence (Calcein AM), while dead cells exhibit red fluorescence (PI).


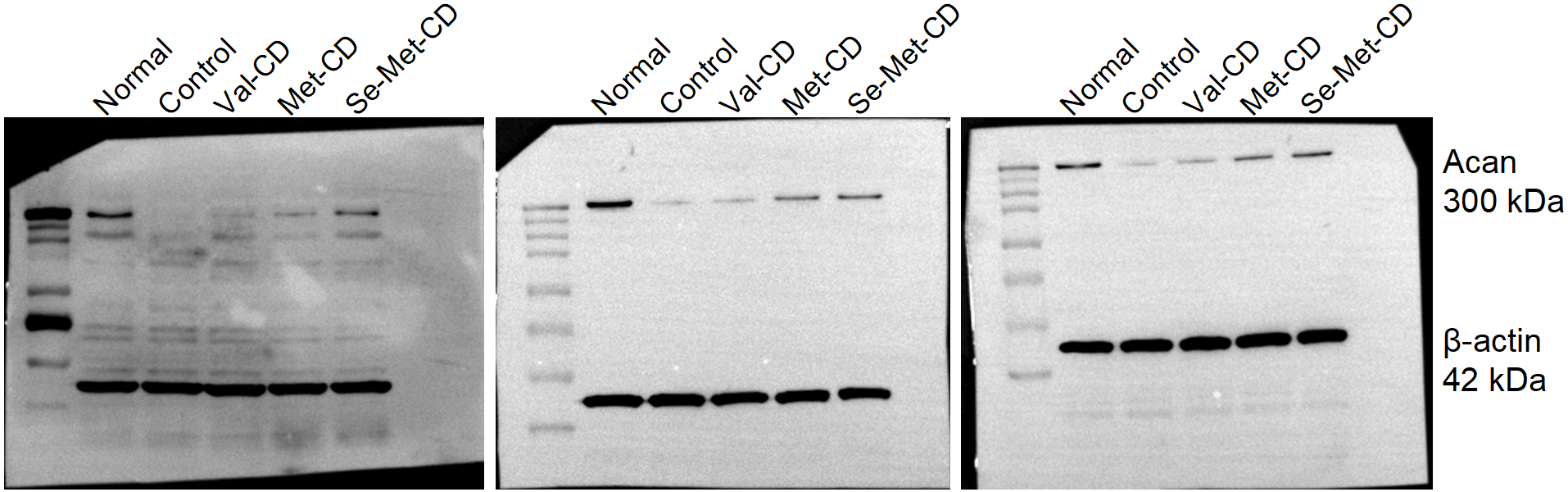


**Figure S13.** Uncropped original blots for Acan. These images are the complete, uncropped Western blots from three independent biological replicates used for the Acan analysis presented in Figure 6B. Molecular weight markers (kDa) are indicated.


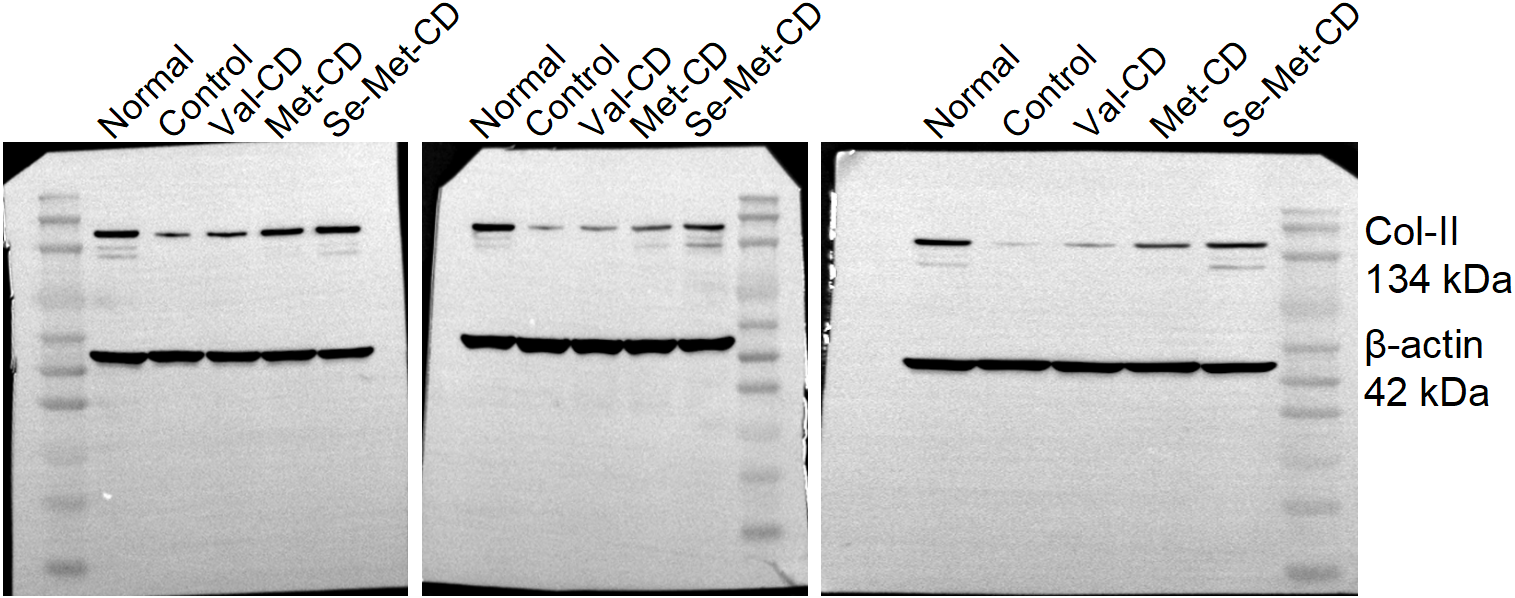


**Figure S14.** Uncropped original blots for Col-II. These images are the complete, uncropped Western blots from three independent biological replicates used for the Col-II analysis presented in Figure 6B. Molecular weight markers (kDa) are indicated.


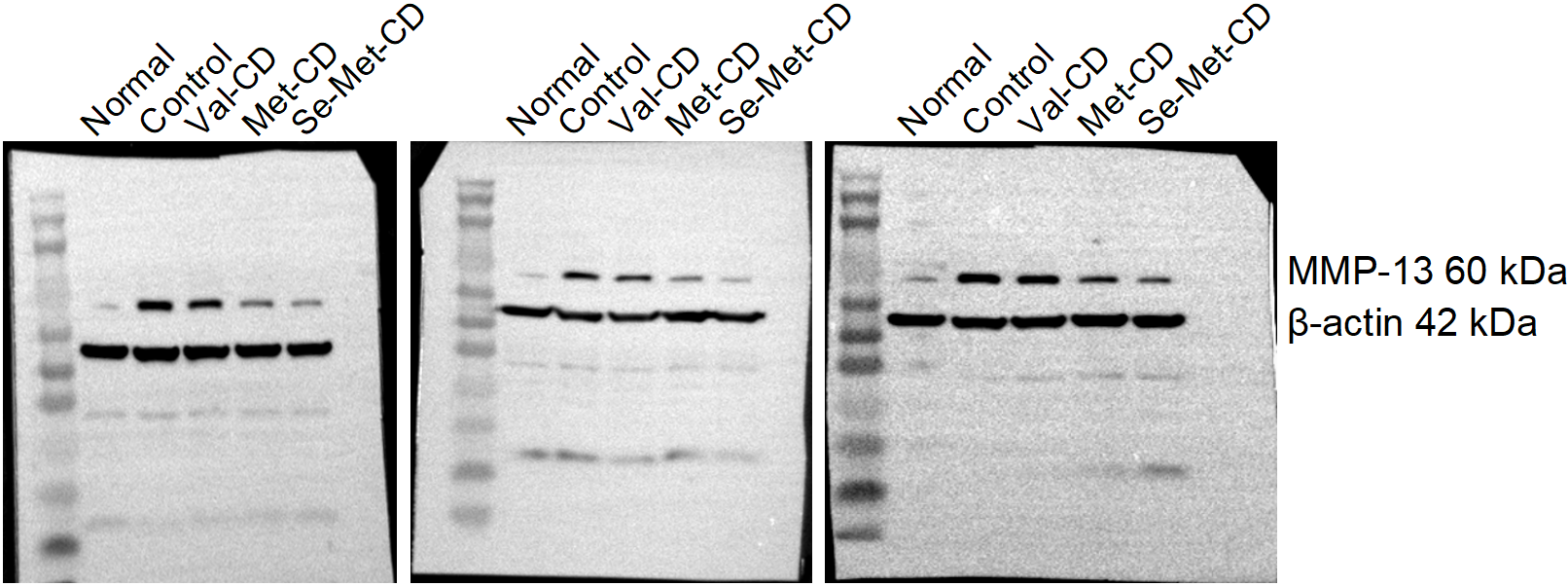


**Figure S15.** Uncropped original blots for MMP-13. These images are the complete, uncropped Western blots from three independent biological replicates used for the MMP-13 analysis presented in Figure 6B. Molecular weight markers (kDa) are indicated.


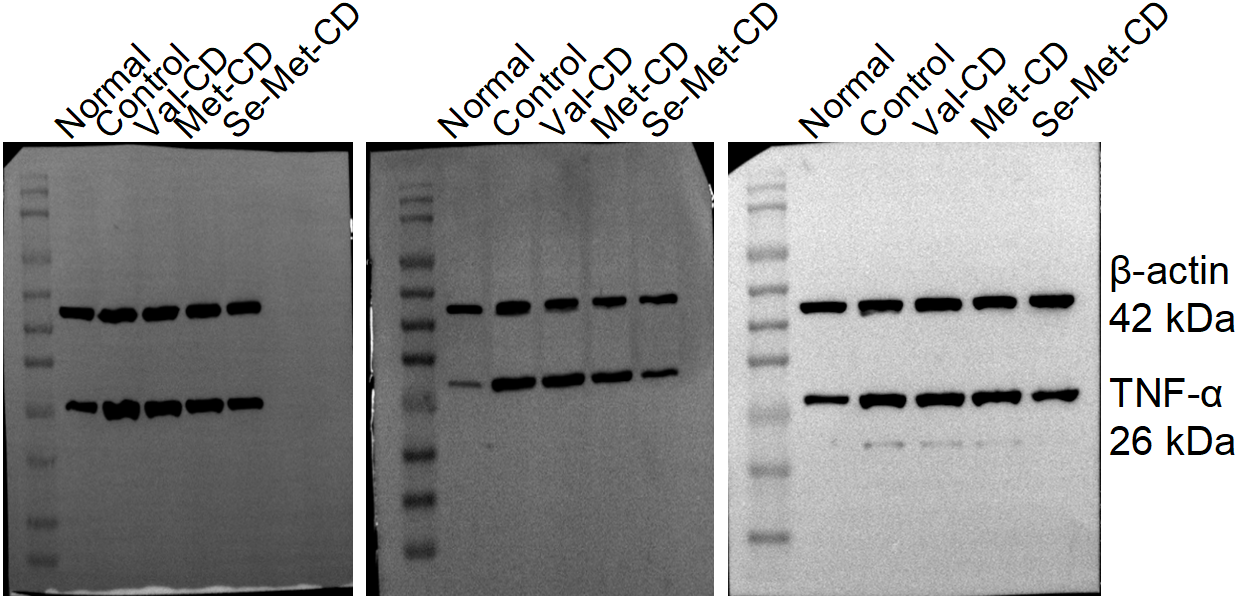


**Figure S16.** Uncropped original blots for TNF-α. These images are the complete, uncropped Western blots from three independent biological replicates used for the TNF-α analysis presented in Figure 6B. Molecular weight markers (kDa) are indicated.


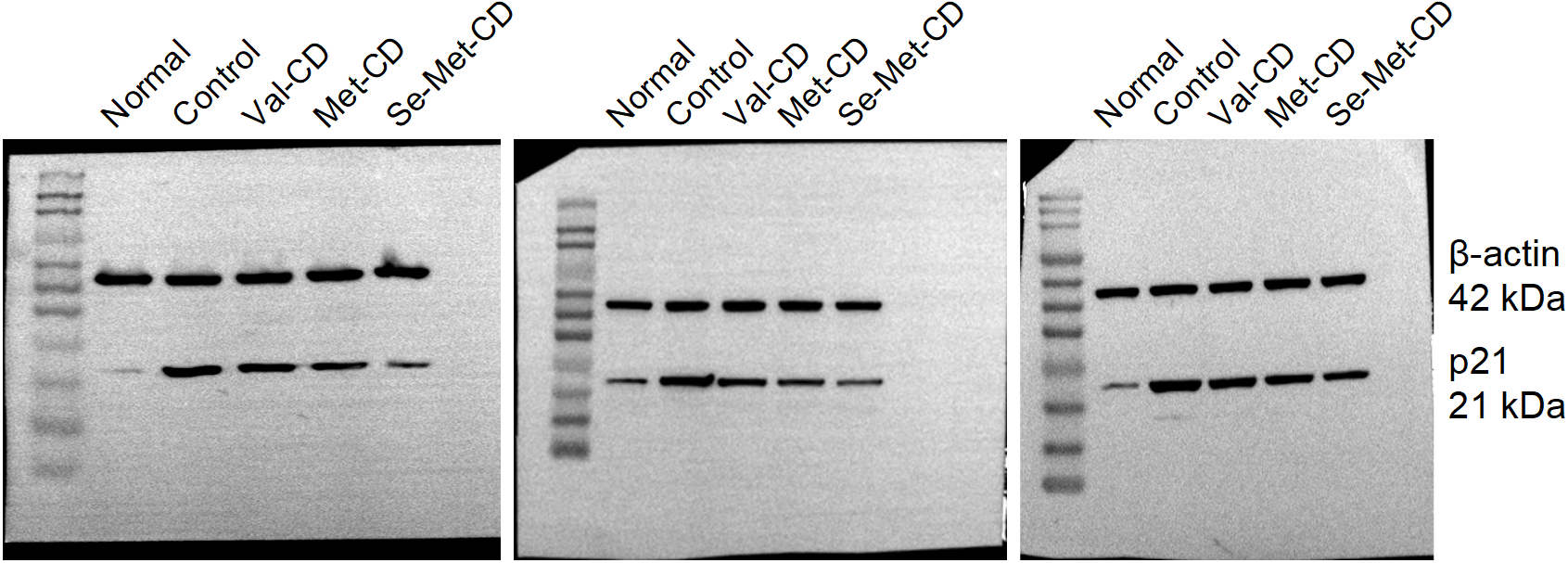


**Figure S17.** Uncropped original blots for p21. These images are the complete, uncropped Western blots from three independent biological replicates used for the p21 analysis presented in Figure 6B. Molecular weight markers (kDa) are indicated.


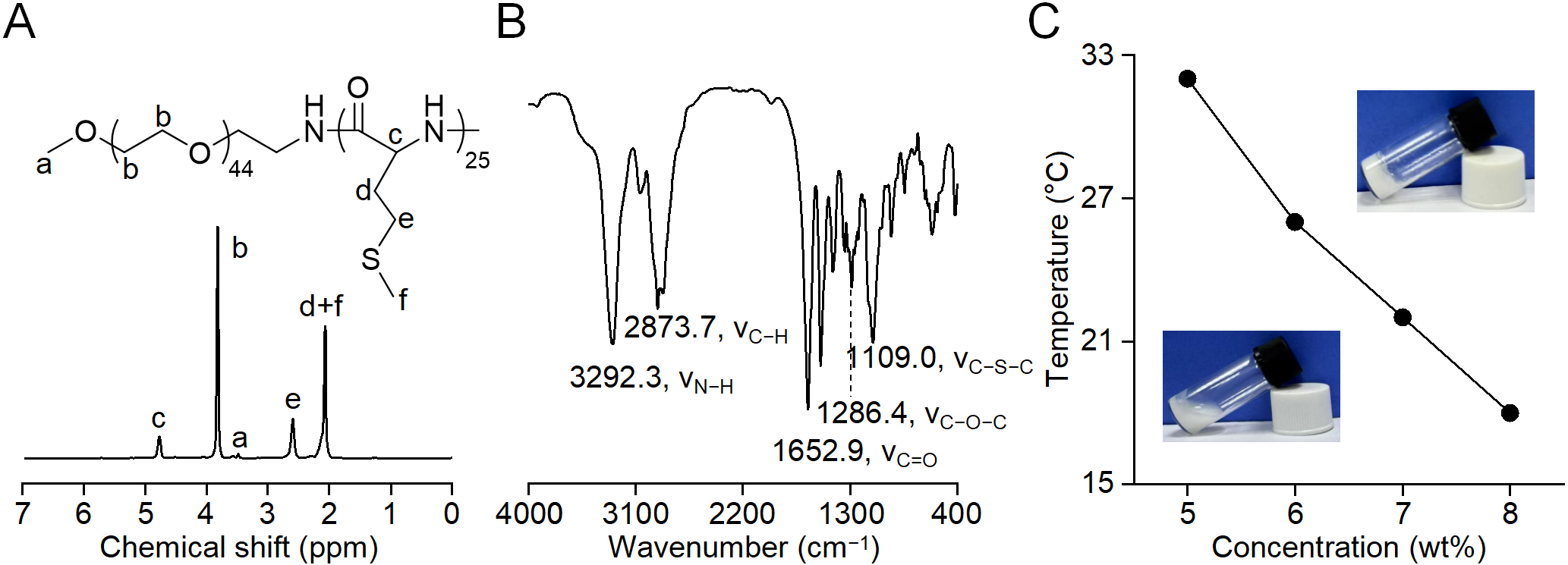


**Figure S18.** Chemical structures and thermo-sensitive gelation properties of mPEG_45_-*b*-PMet_25_ (PM). (A) ^1^H NMR spectrum of PM. (B) FT-IR spectrum of PM. (C) Phase diagram illustrating sol−gel transition temperature of PM hydrogel at various concentrations, with insets showing hydrogel in its sol state (left) and gel state (right).


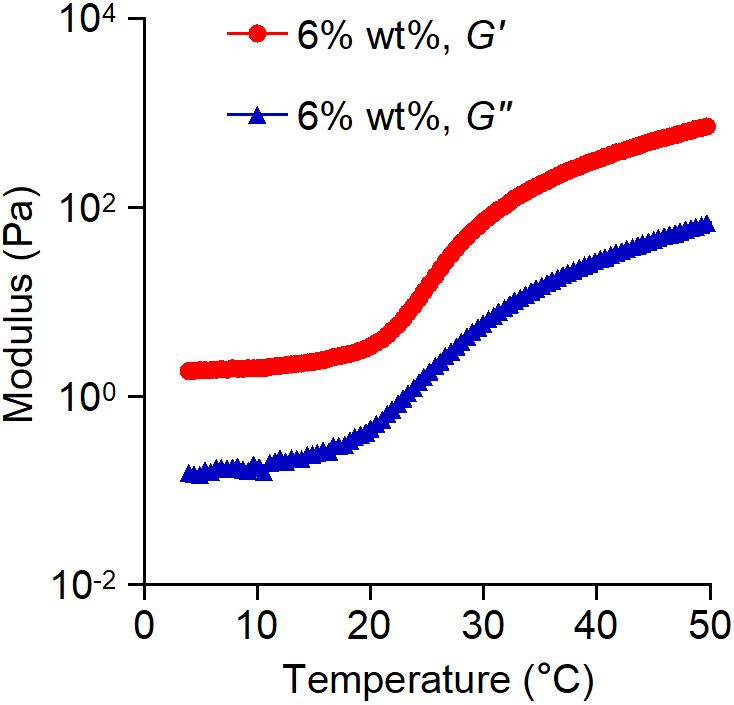


**Figure S19.** Rheological analysis of thermo-sensitive PM hydrogel.


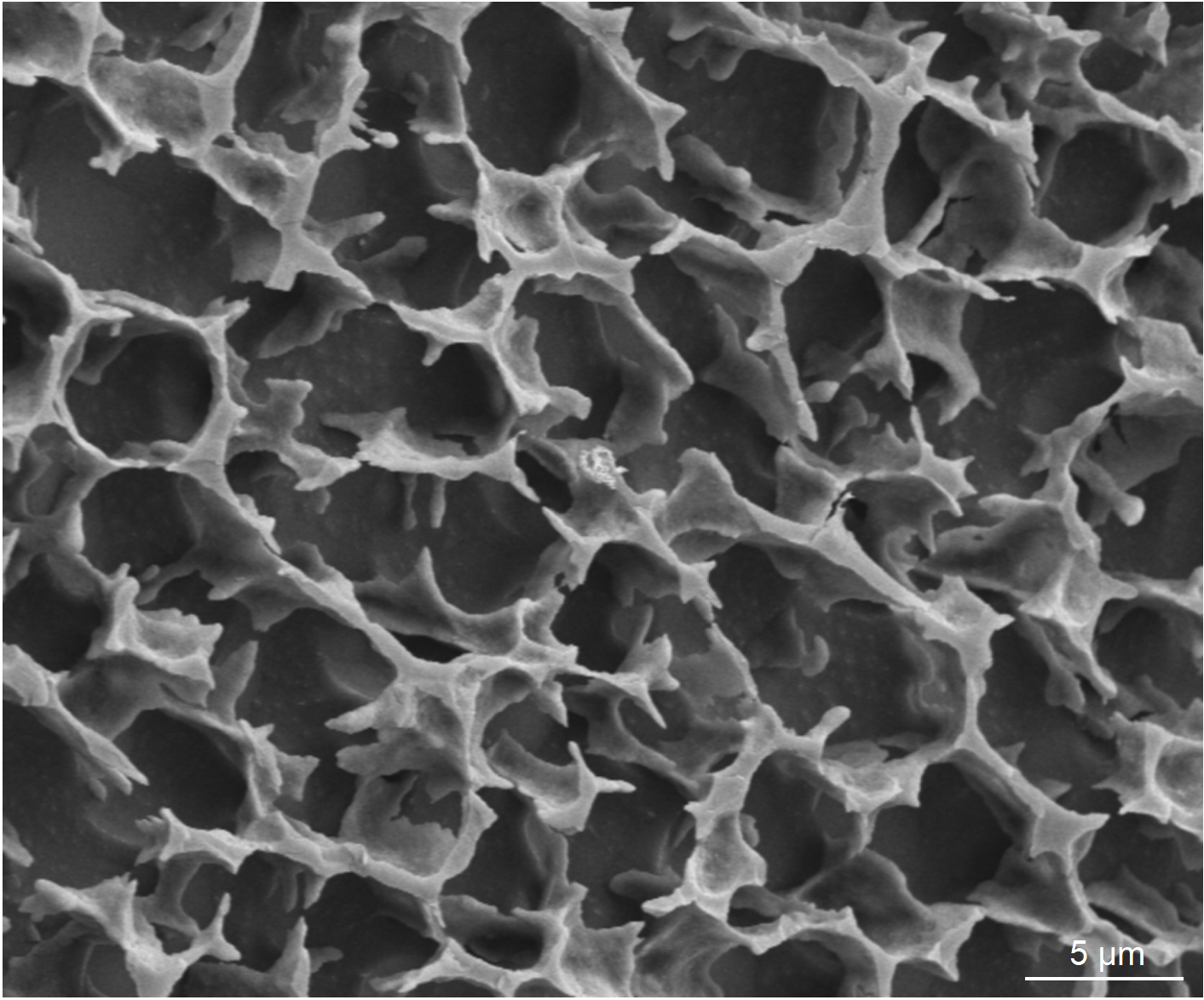


**Figure S20.** Microstructure of PM hydrogel. This representative scanning electron microscopy (SEM) micrograph shows the internal morphology of the lyophilized PM hydrogel.


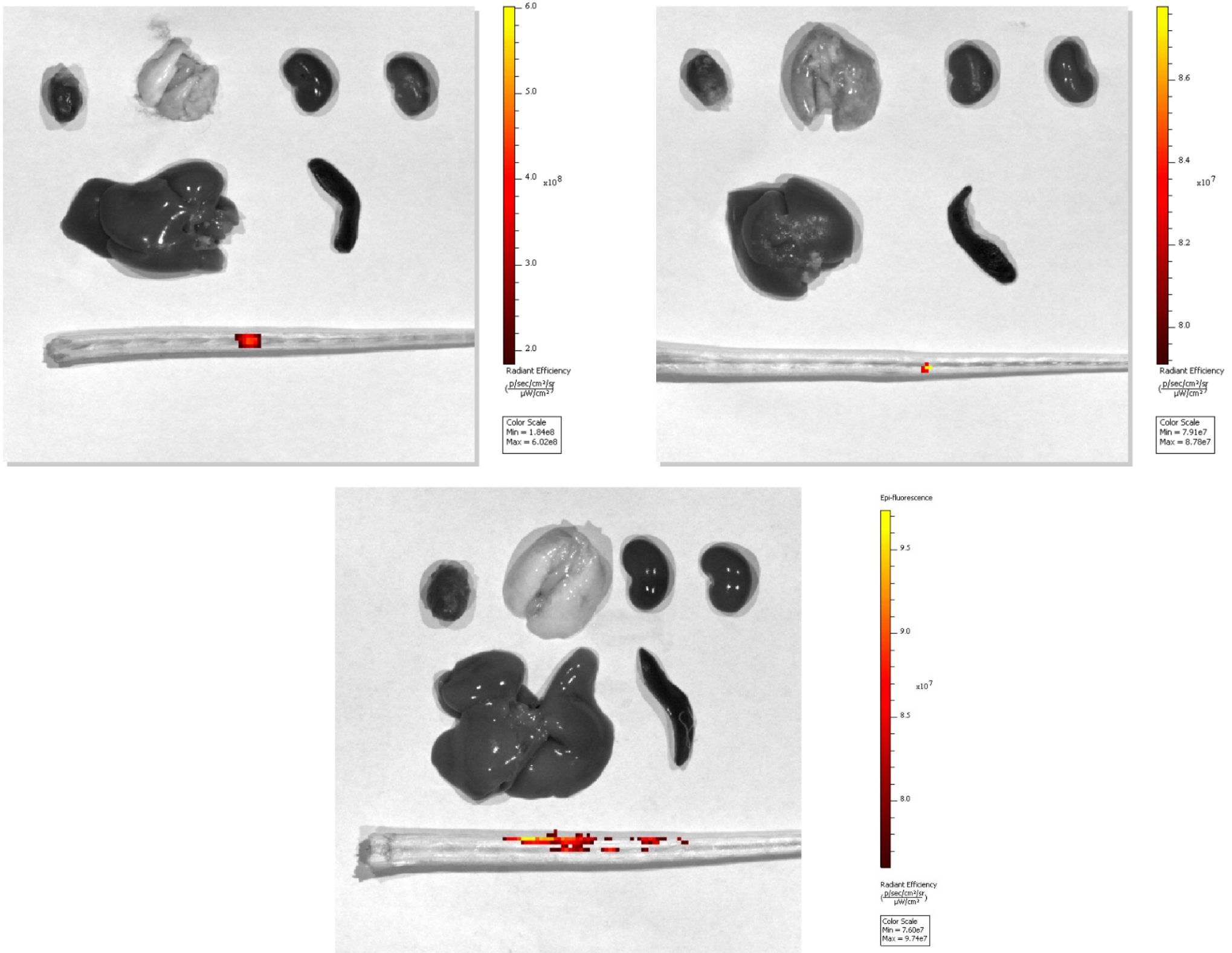


**Figure S21.** Biodistribution of Se-Met-CDs 24 h after local intradiscal injection. Representative *ex vivo* fluorescence images of major organs (heart, lungs, kidneys, liver, spleen) and the injected caudal spine (Injection Site) are shown. Tissues were harvested from rats 24 h after a single intradiscal injection of Cy5.5-labeled Se-Met-CDs, following a whole-body perfusion with PBS to remove blood.


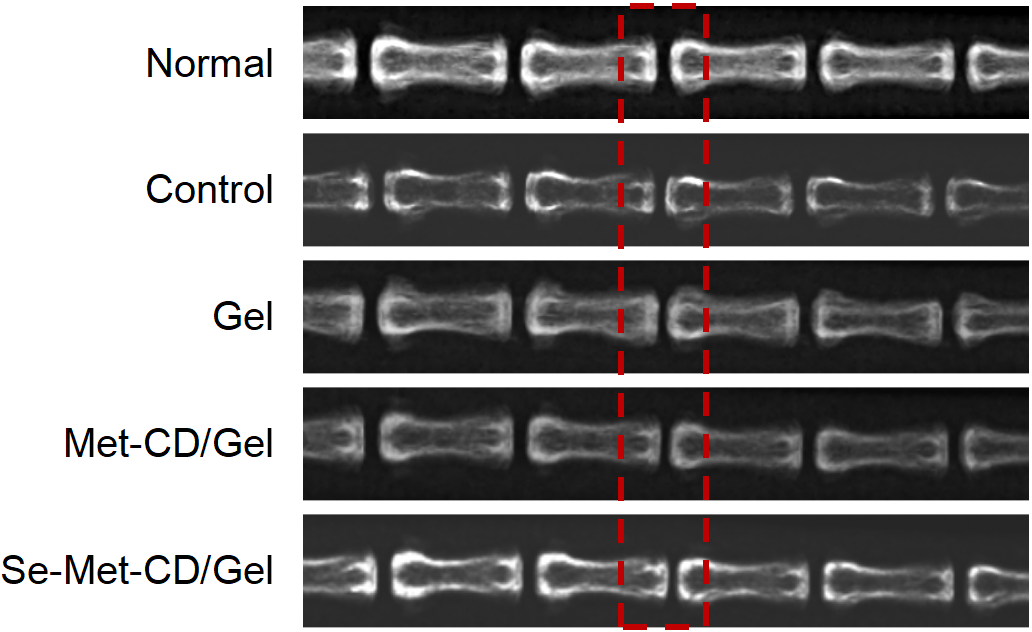


**Figure S22.** Digital radiography (DR) images of rat caudal spines at four weeks post-treatment. Representative DR images for all treatment groups at the 4-week time point. The red boxes indicate the Co7/8 disc space.


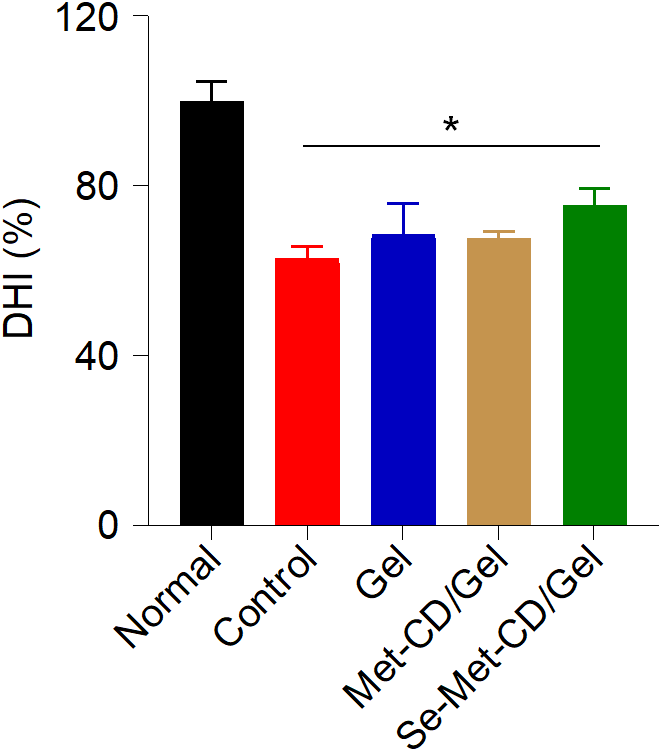


**Figure S23.** Disc height index (DHI) at the 4-week time point. Quantification of DHI (%) from DR images for all treatment groups at four weeks post-operation. Data are represented as the mean ± SD (*n* = 3; **p* < 0.05).


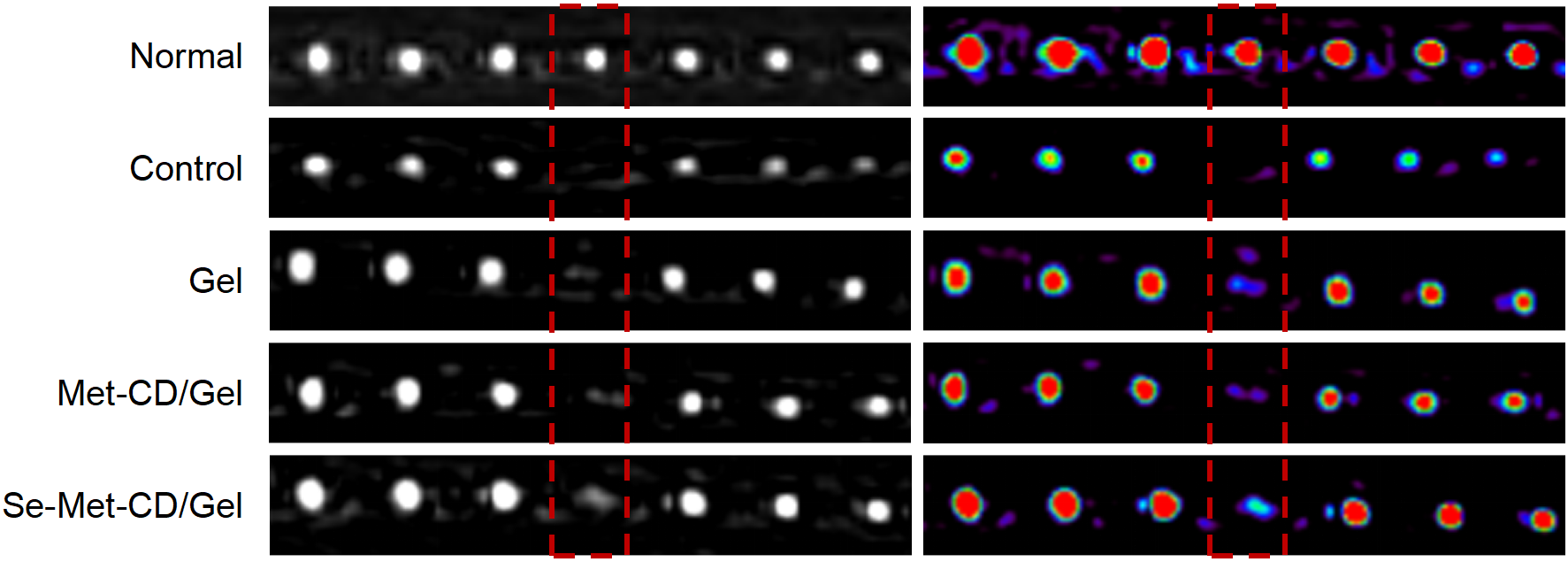


**Figure S24.** T2-weighted MRI images and corresponding pseudocolor maps at four weeks. Representative MRI scans of the Co7/8 intervertebral discs for all treatment groups at four weeks post-operation. The red boxes indicate the Co7/8 disc space. Pseudocolor was applied to the MRI scans to enhance visualization.


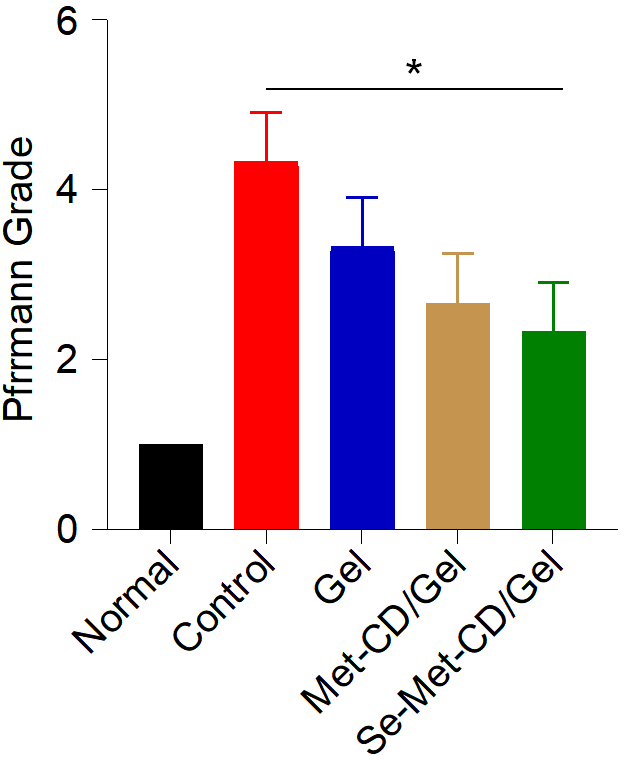


**Figure S25.** Quantitative Pfirrmann scores for all experimental groups, assessed from T2-weighted MRI scans at four-week endpoint.


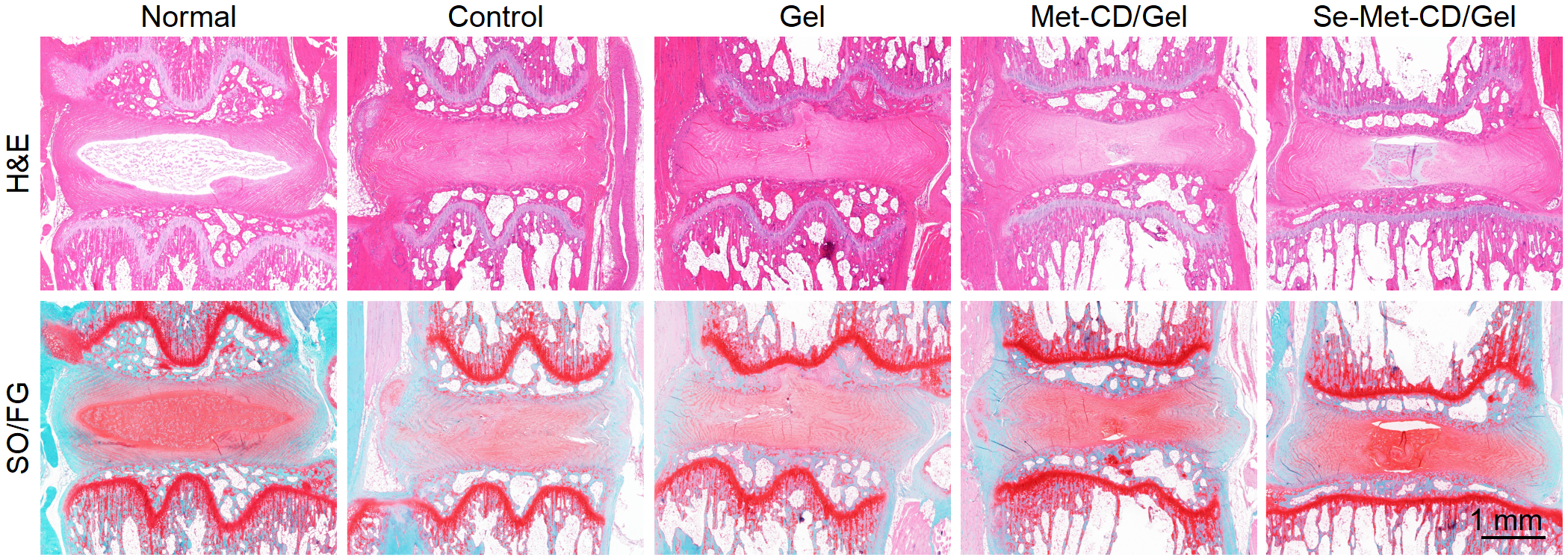


**Figure S26.** H&E and Safranin O/Fast Green staining of Co7/8 disc sections from all groups at four weeks post-operation.


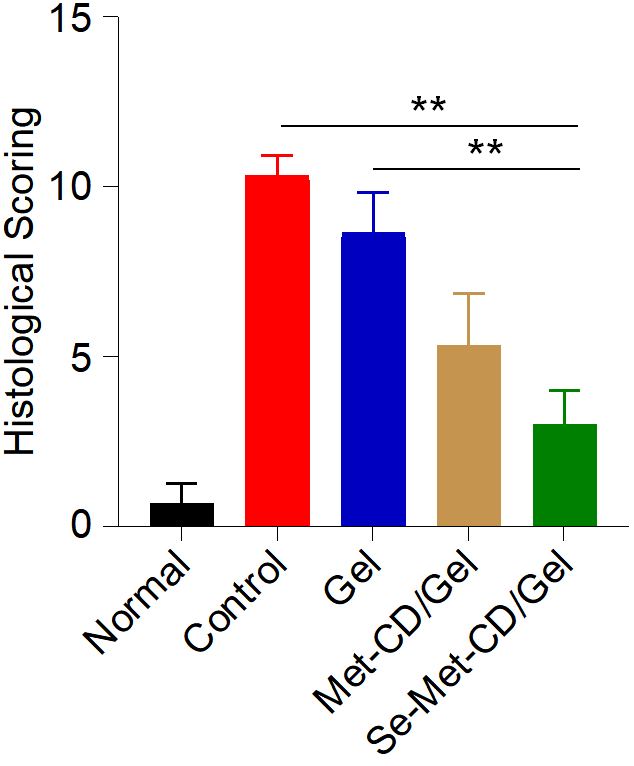


**Figure S27.** Quantitative histological scores of intervertebral discs at four weeks. Histological grading of all experimental groups was performed on the stained sections, where a lower score indicates a healthier disc structure.
